# Proteomic analysis of buccal gland secretion from fasting and feeding lampreys (*Lampetra morii*)

**DOI:** 10.1186/s12953-018-0137-5

**Published:** 2018-05-22

**Authors:** Bowen Li, Meng Gou, Jianmei Han, Xiaofei Yuan, Yingying Li, Tiesong Li, Qi Jiang, Rong Xiao, Qingwei Li

**Affiliations:** 1 School of Life Sciences, Liaoning Normal University, Dalian, 116081 People’s Republic of China; 2 Lamprey Research Center, Liaoning Normal University, Dalian, 116081 People’s Republic of China

**Keywords:** Lampreys, Buccal gland secretion, Fasting, Feeding, Proteomic analysis

## Abstract

**Background:**

Previous studies have shown that lamprey buccal glands contain some regulators related to anticoagulation, nociception, and immune responses due to the blood sucking habit. Regrettably, the protein expression profile in the buccal glands of feeding lampreys has never been reported yet. The present study was performed in order to further identify more proteins which are closely associated with lamprey feeding process.

**Methods:**

2D-PAGE, NanoLC–MS/MS with higher resolution, Ensembl lamprey and NCBI protein databases, as well as western blot was used to compare the proteomics of buccal gland secretion from China northeast lampreys (*Lampetra morii*) which had been fed for 0, 10, and 60 min, respectively.

**Results:**

In the present study, the number of identified protein species in the buccal glands of feeding groups (60 min) was increased significantly, nearly ten times of that in the fasting group. During the feeding stage, novel proteins emerged in the buccal gland secretion of lampreys. According to gene ontology (GO) analysis and function predictions, these proteins were summarized and discussed based on their potential roles during feeding process. Furthermore, some of the identified proteins were confirmed to express during the feeding time of lampreys.

**Conclusion:**

When lampreys attack host fishes to suck blood and flesh, their buccal glands could secrete enough proteins to suppress blood coagulation, nociception, oxidative stress, immune response, as well as other adverse effects encountered during their parasitic lives. The present study would provide clues to clarify the feeding mechanism of the bloodsucking lampreys.

**Electronic supplementary material:**

The online version of this article (10.1186/s12953-018-0137-5) contains supplementary material, which is available to authorized users.

## Background

The jawless lampreys are known as the “living fossils” and considered as ideal animal models to study the vertebrate evolution, development and adaptive immune origin [1–3]. Usually, lampreys live a unique semi-parasitic life cycle: ammocoetes (larvae), parasitic phase lampreys and non-parasitic phase lampreys. Only parasitic phase lampreys could use their sucker-like buccal funnels which are embedded with numerous epidermal teeth to attach to host fishes and suck blood. Due to this blood feeding habit, it is not surprising that lampreys are regarded as bloodsuckers [4]. Similar to the active regulators from salivary glands of the other bloodsuckers, such as leeches, ticks, and bats, lampreys have to secrete active components to counteract the various responses of host fishes during feeding time [5, 6]. Previous studies have shown that the secretion (also called lamphredin) from lamprey buccal glands has anticoagulant and cytolytic properties [4, 7, 8]. However, little is known about the identification and characterization of these active proteins. Until 2007, Xiao and colleagues firstly reported lamprey buccal gland secretion has the fibrinogenolytic property which might block the blood coagulation of host fishes [9]. Different from the multiple components reported in the salivary glands of the other bloodsuckers, only two abundant proteins (buccal gland secretion protein 1, BGSP-1; cysteine-rich buccal gland protein, CRBGP) were detected in the lamprey buccal gland secretion, which might be attributed to the non-feeding habit of non-parasitic adult lampreys (*Lampetra japonica*, *L. japonica*) captured in the previous study [9]. In general, non-parasitic adult lampreys would migrate from ocean to spawn, and since then they feed on nothing. To date, studies on the active proteins in the parasitic lamprey buccal glands are rarely reported. Whether the components of buccal gland secretion are different between non-parasitic and parasitic phase lampreys still remains unknown. With the completion of lamprey genome sequencing, proteomic analysis would become possible to screen active components in the buccal gland secretion of lampreys during fasting and feeding stages [10]. In this study, northeast lampreys (*Lampetra morii, L. morii*) were used to attack host fishes and suck blood. Thus, proteomic characterizations of buccal gland secretion from both fasting and feeding lampreys were analyzed, compared and summarized, which could provide new information on the identification of proteins which are closely associated with lamprey feeding process.

## Methods

### Animals and preparation of lamprey buccal gland secretion

Thirty live northeast lampreys were obtained in December 2014 in Yalu River in Liaoning province of China. The body length of these lampreys was 150–200 mm. The handling of lampreys was approved by the Animal Welfare and Research Ethics Committee of the Institute of Dalian Medical University (Permit number: SYK2004–0029). These lampreys were kept in fresh water at 10 ± 2 °C without feeding for 14 days to avoid the loss of difference in buccal gland components between fasting and feeding stages. And then they were randomly divided into three groups (10 in each group) and kept in the fresh water in the dim light. Subsequently, three groups of lampreys were fed with catfish (*Silurus asotus*) for 0, 10 and 60 min, respectively. After removing the lampreys from the catfish, the buccal glands of these lampreys were dissected and their secretion was immediately collected with a syringe. Finally, the secretion from the buccal glands of each feeding group (*n* = 10) was pooled before analysis.

### Total protein extraction and electrophoresis analysis

The buccal gland secretion was lyophilized by a vacuum freeze dryer (Gold SIM, USA) and homogenized in the lysis buffer containing 7 M urea, 50 mM Tris-HCl (pH 7.4), 50 mM NaCl (Sigma, USA). The homogenates were placed on ice for 0.5 h and then centrifuged (12,000 rpm) at 4 °C for 10 min. The concentration of the supernatant was determined by a Bicinchoninic Acid (BCA) protein assay kit (Beyotime, China) using bovine serum albumin as a standard. At first, the protein samples were loaded on the immobilized pH gradient (IPG) strips with pH range of 3–10. Isoelectric focusing (IEF) was carried out at 0.01 mA/IPG under the following parameters: 250 V, 30 min, linear; 500 V, 30 min, linear; 1000 V, 1 h, rapid; 4000 V, 3 h, linear; 4000 V, 28000 Vh, linear; 500 V, linear. After IEF, the above strips were applied on the 12% sodium dodecyl sulfate-polyacrylamide gel electrophoresis (SDS-PAGE). Electrophoresis was carried out at 5 mA per gel for 1 h, and then at 15 mA per gel for 2 h in PowerPacTM Basic 300 V system (Bio-Rad, USA). When the electrophoresis finished, the gels were stained with Coomassie brilliant blue R-250 for 2 h and destained with 7% acetic acid containing 5% methanol for 12 h. Furthermore, the protein samples were also directly analyzed by 12% SDS-PAGE and Tricine SDS-PAGE respectively, in order to avoid interference induced by BGSP-1 and CRBGP. After staining with Coomassie brilliant blue R-250 for 2 h, the gels were destained in 7% acetic acid containing 5% methanol for 12 h. Subsequently, the SDS-PAGE and Tricine SDS-PAGE gels were respectively cut into 22 and 9 bands on average and transferred to eppendorf tubes.

### In-gel trypsin digestion

All gel bands were further cut into smaller pieces and washed with Milli-Q water (Millipore, USA) at room temperature for 10 min, respectively. Subsequently, the gels were respectively destained with 50 mM ammonium bicarbonate dissolved in acetonitrile (Sigma, USA) at 37 °C for 20 min. After completely destaining and residual detergents removing, the samples were dehydrated in acetonitrile at room temperature for 10 min. When the gel pieces became opaque white, they were reductively alkylated by 10 mM dithiothreitol (Sigma, USA) at 56 °C for 60 min and 50 mM iodoacetamide (Sigma, USA) at room temperature in a darkroom for 45 min, respectively. Next, the gel pieces were digested overnight by trypsin (Sigma, USA) at 37 °C. The peptide mixtures were collected and transferred into new tubes for further Michrom Advance™ nano/cap LC-Q-TOF MS (Bruker, USA) analysis.

### NanoLC–Q-TOF MS analysis

From the resulting solution, 5 μL was initially applied to a 2-cm long (100 μm internal diameter) trap column packed with 5 μm, 200A Magic C18 AQ matrix (Michrom Bioresources, USA) followed by separation on a 15.0-cm long column that was packed with 3 μm, 200A Magic C18 AQ. Samples were loaded onto the trap column at 10000 nL/min, while chromatographic separation occurred at 200 nL/min. Mobile phase A and C are consisted of 0.1% (*v*/v) formic acid in water while mobile phase B is consisted of 0.1% (v/v) formic acid in acetonitrile, and gradient conditions were as follows: 5 to 40% B in 40 min; up to 80% B in 4 min, maintaining this concentration for 10 min. Eluted peptides were directly introduced into a CaptiveSpray Ionization (CSI)-Q-TOF MS (Bruker, USA) for analysis. Dry temperature to 165 °C and capillary voltage to 1500 V. MS1 spectra were acquired from 50 to 2200 m/z at about 20,000 resolution (for m/z 445.1200). For each spectrum, the 5 most intense ions were subjected to Collision Induced Dissociation (CID) fragmentation followed by MS2 analyzer.

### Data analysis

All MS/MS data were analyzed using Compass 1.4, Data Analysis 4.1 bulid 335 (Bruker, USA), and Proteinscape 3.0 (Bruker, USA) for protein search. Ensembl lamprey protein database (www.ensembl.org) and NCBI protein database (www.ncbi.nlm.nih.gov) were used separately to replenish each other for the peptide searching. The search parameters are listed as follows: Fixed Modifications: Carboxymethyl (C) Vari; Modifications: Oxidation (M); Missed Cleavages: 1; Peptide Charge: 1+, 2+ and 3+; Taxonomy: All entries; Peptide Tolerance: 0.1 Da; MS/MS Tolerance: 0.2 Da; Mass: Monoisotopic; Min. Ion Score: 15; Significance: 0.05; Min. Peptide Length: 5. Mascot was used to give every peptide we identified a test of independence, to make sure every peptide’s significance threshold *P* < 0.05. And the distribution of mass error is near zero and most of them are less than 20 ppm (Additional file 1: Figure S1). The data were gathered in the area-proportional Venn diagrams which depicted the variation in number of shared and distinct buccal gland secretion proteins of lampreys which fed for different times.

### Western blot

The protein samples were electrophoresed on 12% SDS-PAGE and then transferred onto the nitrocellulose membranes (Millipore, Germany). After blocking with 5% skim milk, the membranes were incubated with the rabbit anti-human-globin (Sangon Biotech, China, 1:500), the rabbit anti-human-cathepsin D (Sangon Biotech, China, 1:500) and rabbit anti-human-prohibitin 2 (PHB2) (Sangon Biotech, China, 1:500) at 4 °C overnight, respectively. Subsequently, the membranes were washed with PBS for five times, and then incubated with horseradish peroxidase (HRP)-conjugated goat anti-rabbit antibodies (Thermo Fisher Scientific, USA) at a ratio of 1:5000 at room temperature for 2 h. Finally, the membranes were developed with enhanced chemiluminescence kit (Santa Cruz, USA).

## Results

### The number of identified protein species was increased in the buccal gland secretion of lampreys during feeding process

**Fig. 1:**
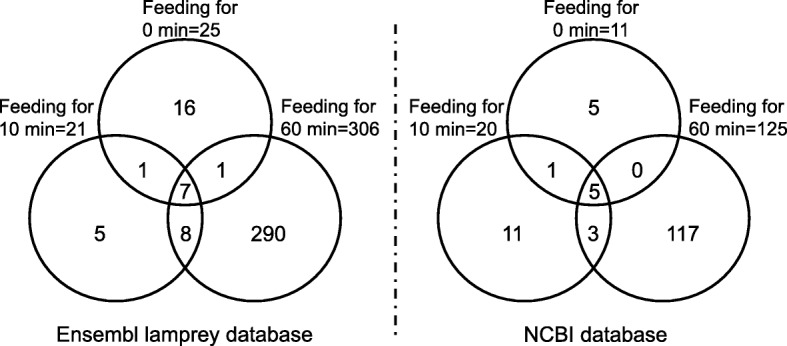
The different number of identified protein species in the buccal gland secretion of lampreys during fasting and feeding stages in Ensembl lamprey and NCBI databases. The number of the identified proteins was summarized from the data in excel (Additional file 1). More proteins were identified in Ensembl lamprey database than that in NCBI database because Ensembl lamprey database contains lots of sequences from lampreys (*Petromyzon marinus*)

Proteomic analysis was performed to evaluate the protein expression profile in the buccal glands of lampreys (*L. morii*) between fasting and feeding stages. According to the Ensembl lamprey database, the number of protein species in the buccal glands of lampreys which had been fed for 0, 10 and 60 min was 25, 21 and 306, respectively (Fig. 1). Only 7 identical proteins including inter-alpha (globulin) inhibitor H5 (ITIH-5, ENSPMAP00000005508), Asp-Glu-Ala-Asp (DEAD) box polypeptide 27 (DDX27, ENSPMAP00000003404), fibronectin leucine rich transmembrane 3 (ENSPMAP00000002471), and myoglobin (ENSPMAP00000005857, ENSPMAP00000005910, ENSPMAP00000001744, ENSPMAP00000005891) existed in the three feeding groups. Apart from the above-mentioned proteins, only 1 (fibronectin leucine-rich repeat transmembrane protein 3, FLRT3, ENSPMAP00000002473), 1 (plasma albumin prepeptide, ENSPMAP00000008289), and 8 (cytochrome P450, ENSPMAP00000008914; melanotransferrin, ENSPMAP00000006073; N-acylsphingosine amidohydrolase, ENSPMAP00000009056; stomatin, ENSPMAP00000008259; prosaposin, ENSPMAP00000008190; titin, ENSPMAP00000002451; protein FAM107B, ENSPMAP00000003872; keratin, ENSPMAP00000007609) proteins were identical when the comparison was made between 0 and 10 min, 0 and 60 min, as well as 10 and 60 min feeding groups. Based on the NCBI database, the number of identified protein species in these three feeding groups was 11, 20 and 125, respectively (Fig. 1). And these three groups only shared 5 identical proteins including plasma albumin (gi|126,143,340), CRBGP (gi|145,046,200), globin (gi|110,825,990), hemoglobin (gi|30,750,169) and pyruvate kinase (PK, gi|313,681,747). Apart from the above-mentioned proteins, only 1 (Deoxygenated hemoglobin, gi|4,389,013), 0, and 3 (LysR family transcriptional regulator, gi|218,461,704; fructose-bisphosphate aldolase, FBA, gi|1,703,239; hemoglobin 2, gi|126,143,345) proteins were identical when the comparison was made between 0 and 10 min, 0 and 60 min, as well as 10 and 60 min feeding groups. Thus, the number of identified protein species in the buccal gland secretion was increased significantly when lampreys fed on host fishes for 60 min.

### The buccal glands of lampreys could generate more proteins to participate in the feeding process

**Fig. 2:**
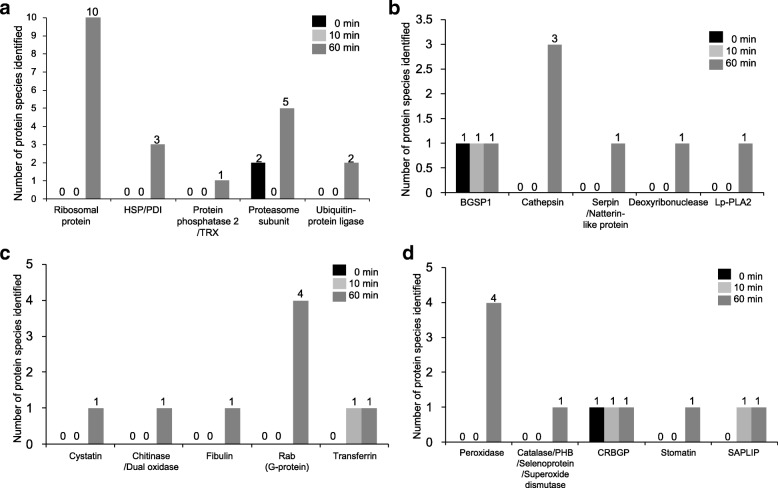
The number of identified proteins which might be associated with feeding process of lampreys. The number of identified ribosomal protein, heat shock protein (HSP), protein disulfide isomerase (PDI), protein phosphatase 2, thioredoxin (TRX), proteasome subunit and ubiquitin protein ligase in the 0 min, 10 min, and 60 min feeding groups was shown in panel **a**. The number of identified proteins related to anticoagulation including BGSP-1, cathepsin, serpin, natterin-like protein, deoxyribonuclease I and lipoprotein-associated phospholipase A2 (Lp-PLA2) group VII was shown in panel **b**. The number of cystatin, chitinase, dual oxidase, fibulin, Rab (G-protein) and transferrin was shown in panel **c**. The number of identified peroxiredoxin, catalase, prohibitin 2 (PHB2), superoxide dismutase, selenoprotein, cysteine-rich buccal gland protein (CRBGP), stomatin and saposin-like protein (SAPLIP) was shown in panel **d**

**Fig. 3:**
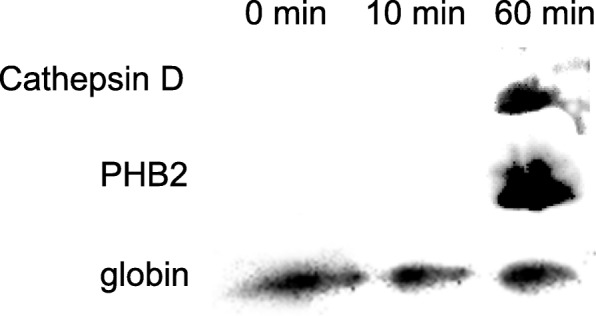
The expression of cathepsin D, PHB2 and globin in the buccal glands of lampreys feeding for 0 min, 10 min and 60 min, respectively. Globin was expressed all the time and used as a control; while cathepsin D and PHB2 were expressed only at 60 min feeding group

According to gene ontology (GO) analysis, the identified proteins in these three groups were classified based on their characterizations, including biological process, molecular function and cellular component (Additional file 1: Table S1). Although the classification and its ratio are different in some extent between the two databases, more novel proteins appeared when lampreys fed on catfish for 60 min. From GO analysis among the three groups, proteins which might play important roles during feeding stage of lampreys were chosen and summarized. As shown in Table 1, these proteins were mainly classified into 9 groups: the first group is related to protein synthesis, modification, and degradation; the second group includes proteins to regulate blood flow, blood pressure, and vascular contractility; the third and fourth groups are composed of anticoagulants and immune regulators, respectively; the fifth and sixth groups contain proteins which might play important roles in nociception and antioxidation; the seventh and eighth groups are associated with anti-angiogenesis and haemolysis; the last group contains proteins to participate in the glycolysis and signal transduction. In addition, the number of these identified proteins was shown in Fig. 2. Among the various identified proteins, about 94% proteins newly emerged in the 60 min feeding group. The above results indicated that buccal gland secretion from the feeding lampreys (60 min) contained a variety of active proteins which could help lampreys counteract the adverse responses generated from the host fishes during their attachments. In order to further confirm the newly emerged proteins in the 60 min feeding group, western blot was used to analyze the expression of cathepsin D and PHB2. As shown in Fig. 3, both cathepsin D and PHB2 were found expressed in the buccal glands of lampreys fed for 60 min; while neither cathepsin D nor PHB2 was detected in the 0 and 10 min feeding groups.

**Table 1:** Summarization of identified proteins which might be associated with feeding process of lampreys

No.	Protein functions	Protein names
1	Protein synthesis, modification, and degradation	40S ribosomal protein S9/S15a/S20/S25/S26; 60S ribosomal protein L11/L14/L18/L21/L28; Proteasome non-ATPase subunit 12 (26S)/ATPase subunit 2 (26S); β2/β3/β5/Y/non-ATPase subunit 6 (26S); Ubiquitin-conjugating enzyme E2O/E3; Heat shock protein (HSP) 5/90α/90β; Protein disulfide isomerase (PDI) family A, member 3; Thioredoxin (TRX) domain containing 17; Protein phosphatase 2, regulatory subunit B, δ
2	Blood flow, blood pressure, and vascular contractility	Angiotensin II receptor (type 1a); Serine carboxypeptidase 1; Cystathionine β-synthase; Ryanodine receptor 2
3	Anticoagulation	BGSP1; Cathepsin D; Deoxyribonuclease I; Serpin; Natterin-like protein; Lipoprotein-associated phospholipase A2 (Lp-PLA2)
4	Immune regulation	Cathepsin L/Z/D; Cystatin F; Chitinase domain containing 1; Dual oxidase Melanotransferrin/transferrin-a; Rab 1A/2A/11a/29; Serpin
5	Nociception	CRBGP; Stomatin
6	Antioxidation	Peroxidase (Glutathione peroxidase 2; Peroxiredoxin 2/3/TSA1); Selenoprotein 15; TRX domain containing 17; Catalase; Prohibitin 2; Superoxide dismutase 2
7	Anti-angiogenesis	Ubiquinol-cytochrome c reductase binding protein (UQCRB); CRBGP; Fibulin 5; Cystatin
8	Tissue lysis	Saposin
9	Glycolysis and signal transduction	Cytochrome P450; Fructose-bisphosphate aldolase (FBA); 14-3-3 protein; Triosephosphate isomerise; Glyoxalase; Pyruvate kinase (PK)

## Discussion

In the past decades, proteomic studies on anopheles [11], ticks [12], spiders [13] and snakes [14] have also been extensively reported, which provided valuable information on disease prevention and novel drug development. At present, database is the key factor to affect the number of identified protein species through proteomic analysis. Regrettably, the sequences from lampreys are far fewer than that from humanbeing, mice, rats, and even zebra fishes in SwissProt and NCBI databases. To date, SwissProt which is the best annotated protein database, possesses only 67 reviewed protein sequences from lampreys (*Petromyzon marinus*). Also, there are only 4412 sequences from lampreys in NCBI database, which contains lots of repeat or false sequences. 2013, the genome of lampreys was sequenced and assembled [10]. And 14,000 sequences from lampreys were submitted to Ensembl database (www.ensembl.org). In the present study, both SwissProt and NCBI databases are not big enough for protein searching. Thus, we use the two databases to complement each other in order to identify more proteins. Although lamprey proteins could be identified through NCBI database, some of them were identified as proteins from the other species due to the homology. Also, the number of identified proteins was fewer than that in Ensembl database. This is different from the results obtained from Ensembl lamprey database.

In the present study, 2D-PAGE was firstly used to analyze the composition of proteins in the buccal glands of lampreys which have been fed for 10 min and 60 min, respectively. As shown in Additional file 1: Figure S2, there were almost no differences on the 2D-gels between the two feeding groups probably due to the affection induced by the high content of BGSP-1 and CRBGP. Thus, we applied the protein samples directly on 12% SDS-PAGE and Tricine SDS-PAGE, and then digested them with trypsin in gels in order to detect more proteins with relatively lower content compared with that of BGSP-1 and CRBGP.

**Fig. 4:**
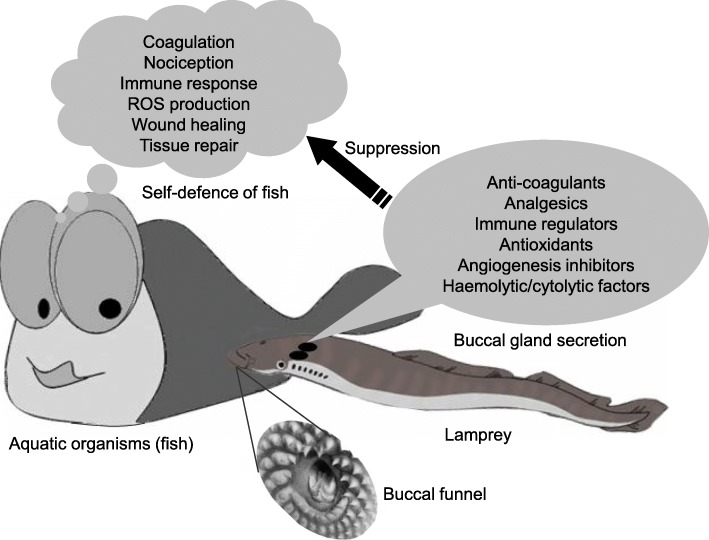
Lampreys have to evolve various strategies to subvert the adverse responses generated by host fishes. When lampreys feed on host fishes, novel proteins related to anticoagulation, analgesia, immune regulation, antioxidantion, anti-angiogenesis, and cytolysis were emerged in their buccal glands to suppress the adverse responses generated from host fishes

According to our proteomic analysis, the expression profile of buccal gland secretion from fasting and feeding lampreys was closely associated with their blood sucking habit. Similar to the other blood suckers, lamprey buccal glands could generate lots of proteins to counteract the adverse responses encountered during feeding time (Fig. 4). Compared with the fasting group, the number of identified protein species was increased significantly in the 60 min feeding group, nearly ten times of that in the fasting group, suggested that the feeding habit of lampreys determined the protein expression profile in their buccal glands. Furthermore, the relatively fewer protein species found in the fasting group are consistent with the non-feeding characteristics of lampreys at spawn stage. Compared with the fasting group, the identified protein species number in the 10 min feeding group was not changed obviously, which indicated that lampreys need relatively more time to synthesize enough proteins to inject into host fishes through the ducts. As we all know, the generation of novel proteins usually need two steps: transcription (from DNA to mRNA) and translation (from mRNA to proteins). 10 min might not be enough to complete these two steps. Thus, peroxidase, cathepsin D, as well as the other proteins only appeared in the 60 min feeding group.

According to our western blot analysis, both cathepsin D and PHB2 were detected in the buccal glands of 60 min feeding group, and were not detected in the 0 and 10 min feeding groups. This is consistent with our proteomic data which indicated the accuracy of our analysis. In the present study, we used the rabbit anti-human cathepsin D and anti-human PHB2 antibodies to recognize lamprey cathepsin D and PHB2 probably due to the highly conserved domains in these two proteins. As we all know, not all commercial antibodies were suitable for the proteins originated from lampreys. To date, there are no commercial antibodies for lampreys specially. Thus, we just used these two antibodies to verify the proteomic analysis in the present study. Furthermore, previous studies showed that lamprey cathepsin D and PHB2 were associated with anti-coagulation, immune regulation, and antioxidation, which could suppress the coagulation, immune response, and oxidative stress generated from host fishes effectively [15, 16].

Among the identical proteins shared in the three groups, CRBGP has been reported to inhibit contraction of rat-tail arterial smooth muscle, voltage-gated sodium and potassium channels, neutrophil migration, and angiogenesis, which suggested that CRBGP could block the vasodilatation, nociception, inflamation and wound healing processes of host fishes [17–20]. Besides CRBGP, BGSP-1 shared in these three groups was reported to degrade fibrinogen, indicated BGSP-1 could suppress the blood coagulation of host fishes [9]. Although other proteins including ITIH-5, DDX27, fibronectin leucine rich transmembrane 3, myoglobin, globin, hemoglobin and PK were also found in the three feeding groups, their biological functions have not been reported yet and still need further studies.

When lampreys attacked fishes in the marine, their buccal glands should initiate protein synthesis and modulation system to ensure the production of active proteins to counteract the adverse effects. In our study, the number of ribosomal proteins (Eukaryotes), heat shock protein (HSP), protein disulfide isomerase (PDI), protein phosphatase 2 and thioredoxin (TRX) was increased significantly in the buccal glands of 60 min feeding group (Fig. 2a). And these proteins would cooperate together to provide conditions for the translation, folding and modification. Meanwhile, ubiquitin-proteasome system was also activated in the buccal glands of feeding lampreys (60 min), indicated the unneeded or damaged proteins would be degraded in a non-lysosomal pathway [21]. Furthermore, the secretion was drawn from the buccal glands of lampreys with a syringe. The cells in the buccal glands might be drawn with the secretion at the same time. This might lead to the identification of intracellular proteins. Actually, it is very difficult for us to obtain the secreted liquid from the buccal glands because the feeding is happened in the freshwater or seawater.

Similar to the other blood suckers, lampreys also have to generate novel proteins to suppress the blood coagulation cascade of host fishes (Fig. 2b). According to our previous studies, both BGSP-1 and cathepsin D were proved to be able to degrade fibrinogen [9, 15]; while serpin from *Lampetra fluviatilis* was reported to have anti-FXa activity [22]. This meant that besides BGSP-1, cathepsin D and serpin could be generated in the buccal glands of feeding lampreys to prevent blood from clotting effectively. In addition, a natterin-like protein was identified in the buccal glands of feeding lampreys (60 min). Very recently, natterin from *Thalassophryne nattereri* fish venom was reported to degrade kininogen and kininogen-derived peptides in the same manner as tissue kallikrein [23, 24]. Whether natterin-like protein from lamprey buccal glands might also exhibit the anti-coagulant activities as natterin from fish need further studies. Furthermore, recent studies have reported that both deoxyribonuclease I and lipoprotein-associated phospholipase A2 (Lp-PLA2) group VII were also associated with anti-coagulation [25, 26], while their roles in lamprey buccal glands still need further studies.

During the feeding time, lampreys have to subvert the immune monitoring from host fishes and microorganism infection from environments. Among the newly identified immune regulators, cathepsin D was reported to associate with immune responses because its expression level in buccal glands was significantly increased after the lampreys were stimulated with *Escherichia coli* or *Staphylococcus aureus* [15]. This is consistent with the cathepsin D from the other blood suckers [27]. Besides cathepsin D, cystatin and serpin were also detected in the buccal glands of feeding lampreys (Fig. 2b and c). Although the immunologic characteristics of cystatin and serpin in lampreys have not been reported yet, cystatin and serpin from the other blood suckers had been found to interfere with lymphocyte and macrophage responsiveness, proinflammatory cytokines production, antigen processing and presentation, as well as phagocytosis [28–31]. At present, the biological functions of cystatin in lampreys are still studied in detail. Besides the above proteins, other regulators including chitinase domain containing 1, dual oxidase, fibulin, RAB29 (member RAS oncogene family), and transferrin were also identified in the buccal glands of feeding lampreys and their roles in immune response need further studies (Fig. 2c).

In the feeding stage, lampreys have to secrete antioxidants to suppress damage induced by reactive oxygen species (ROS) from metabolic processes and immune defenses of hosts (Fig. 2d) [32, 33]. 2010, peroxiredoxin 2 was cloned from buccal glands of lampreys and shown to remove H_2_O_2_ and protect DNA from oxidative injury [34]. In this study, three peroxiredoxins which belong to peroxidase family were also detected in the buccal gland secretion of feeding lampreys, indicated that they participate in the feeding process of lampreys. In addition to peroxiredoxins, PHB2, catalase and superoxide dismutase 2 were also identified in the feeding lampreys (60 min). These antioxidants have been proved to enhance the oxidative stress tolerance in Chang liver (CHL) cells significantly, regulate ferritin concentration and remove harmful hydroxyl radicals, meant that these antioxidants are also associated with the feeding process of lampreys [16, 35].

When lampreys use their epidermal teeth to attack host fishes, nociceptive responses would be generated ineluctably and felt evidently by the hosts. Do lampreys only use CRBGP to make hosts lose nociceptive responses and keep them sucking blood continuously? In the buccal glands of feeding lampreys, another ion channel regulator stomatin was also identified (Fig. 2d). Regretably, its functions in lampreys still remain unknown.

As lampreys attack host fishes, they might try to destroy the tissues of fishes. In the present study, a saposin-like protein (SAPLIP) also appeared in both 10 min and 60 min feeding lampreys (Fig. 2d). 2002, a SAPLIP family member cloned from *Clonorchis sinensis* exhibited haemolytic activity toward rabbit erythrocytes [36, 37]. Further studies need to be conducted to figure out whether the SAPLIP in buccal glands of feeding lampreys could also exhibit the property on cell lysis or not.

## Conclusion

In the present study, the proteomic composition in the buccal gland secretion of fasting and feeding lampreys was analyzed and compared for the first time, which could provide important clues for the identification of valuable proteins present in the buccal glands. Although the characterizations of most valuable proteins in lampreys have not been reported yet, their biological functions would be studied in the near future. To summarize, lampreys have to evolve various strategies to generate novel proteins related to anticoagulation, analgesia, immune suppression, antioxidation, anti-angiogenesis, haemolysis and cytotoxicity to subvert the adverse responses of host fishes during their feeding time (Fig. 4).

## Additional file

Additional file 1: **Figure S1.** The distribution of mass errors is near zero and most of them are less than 20 ppm, which means the mass accuracy of the MS data fits the requirement and has a good QC validation of MS data. **Figure S2.** The protein composition of buccal gland secretion from lampreys fed for 10 min and 60 min was detected by 2D-PAGE. **Table S1.** The distribution of identified proteins on biological process (A), molecular function (B) and cellular component (C) during fasting and feeding stages. The top ten terms on biological process, molecular function, and cellular component were listed. Excel The identified protein species in the buccal gland secretion of lampreys during fasting and feeding stages in Ensembl lamprey and NCBI databases. (PDF 927 kb)

## Abbreviations

BCA: Bicinchoninic acid
BGSP-1: Buccal gland secretion protein 1
CHL: Chang liver
CID: Collision Induced Dissociation
CRBGP: cysteine-rich buccal gland protein
CSI: CaptiveSpray Ionization
DDX27: DEAD (Asp-Glu-Ala-Asp) box polypeptide 27
FBA: fructose-bisphosphate aldolase
FLRT3: Fibronectin leucine-rich repeat transmembrane protein 3
GO: Gene ontology
HRP: Horseradish peroxidise
HSP: Heat shock protein
IEF: Isoelectric focusing
IPG: Immobilized pH gradient
ITIH-5: Inter-alpha (globulin) inhibitor H5
*L. japonica*: *Lampetra japonica*
*L. morii*: *Lampetra morii*
Lp-PLA2: Lipoprotein-associated phospholipase A2
PDI: Protein disulfide isomerase
PHB2: Prohibitin 2
PK: Pyruvate kinase
ROS: Reactive oxygen species
SAPLIP: Saposin-like protein
SDS-PAGE: Sodium dodecyl sulfate-polyacrylamide gel electrophoresis
TRX: Thioredoxin

## References

[1] Forey P, Janvier P (1993). Agnathans and the origin of jawed vertebrates. Nature.

[2] Nikitina N, Bronner-Fraser M, Sauka-Spengler T (2009). The sea lamprey *Petromyzon marinus*: a model for evolutionary and developmental biology. Cold Spring Harb Protoc.

[3] Pancer Z, Amemiya CT, Ehrhardt GR, Ceitlin J, Gartland GL, Cooper MD (2004). Somatic diversification of variable lymphocyte receptors in the agnathan sea lamprey. Nature.

[4] Lennon RE (1954). Feeding mechanism of the sea lamprey and its effect on host fishes. Fish Bull U S Dep Interior.

[5] Zavalova LL, Basanova AV, Baskova IP (2002). Fibrinogen-fibrin system regulators from bloodsuckers. Biochemistry (Mosc).

[6] Basanova AV, Baskova IP, Zavalova LL (2002). Vascular-platelet and plasma hemostasis regulators from bloodsucking animals. Biochemistry (Mosc).

[7] Baxter EW (1956). Observations on the buccal glands of lampreys (Petromyzonidae). Proc Zool Soc Lond.

[8] Gage SH, Gage-Day M (1927). The anti-coagulating action of the secretion of the buccal glands of the lampreys (*Petromyzon, Lampetra and Entosphenus*). Science.

[9] Xiao R, Li QW, Perrett S, He RQ (2007). Characterisation of the fibrinogenolytic properties of the buccal gland secretion from *Lampetra japonica*. Biochimie.

[10] Smith JJ, Kuraku S, Holt C, Sauka-Spengler T, Jiang N, Campbell MS, Yandell MD, Manousaki T, Meyer A, Bloom OE, et al. Sequencing of the sea lamprey (*Petromyzon marinus*) genome provides insights into vertebrate evolution. Nat Genet. 2013;45(4):415–21. 421e1–2 10.1038/ng.2568 PMC3709584 23435085

[11] Jariyapan N, Roytrakul S, Paemanee A, Junkum A, Saeung A, Thongsahuan S, Sor-suwan S, Phattanawiboon B, Poovorawan Y, Choochote W (2012). Proteomic analysis of salivary glands of female *Anopheles barbirostris* species A_2_ (Diptera: Culicidae) by two-dimensional gel electrophoresis and mass spectrometry. Parasitol Res.

[12] Di Venere M, Fumagalli M, Cafiso A, De Marco L, Epis S, Plantard O, Bardoni A, Salvini R, Viglio S, Bazzocchi C (2015). *Ixodes ricinus* and its endosymbiont *Midichloria mitochondrii*: a comparative proteomic analysis of salivary glands and ovaries. PLoS One.

[13] Palagi A, Koh JM, Leblanc M, Wilson D, Dutertre S, King GF, Nicholson GM, Escoubas P (2013). Unravelling the complex venom landscapes of lethal Australian funnel-web spiders (Hexathelidae: Atracinae) using LC-MALDI-TOF mass spectrometry. J Proteomics.

[14] Luna MS, Valente RH, Perales J, Vieira ML, Yamanouye N (2013). Activation of *Bothrops jararaca* snake venom gland and venom production: a proteomic approach. J Proteome.

[15] Xiao R, Zhang Z, Wang H, Han Y, Gou M, Li B, Duan D, Wang J, Liu X, Li Q (2015). Identification and characterization of a cathepsin D homologue from lampreys (*Lampetra japonica*). Dev Comp Immunol.

[16] Li T, Wang Y, Gao Y, Li Q (2015). Identification and characterisation of the anti-oxidative stress properties of the lamprey prohibitin 2 gene. Fish Shellfish Immunol..

[17] Ito N, Mita M, Takahashi Y, Matsushima A, Watanabe YG, Hirano S, Odani S (2007). Novel cysteine-rich secretory protein in the buccal gland secretion of the parasitic lamprey, *Lethenteron japonicum*. Biochem Biophys Res Commun.

[18] Chi S, Xiao R, Li Q, Zhou L, He R, Qi Z (2009). Suppression of neuronal excitability by the secretion of the lamprey (*Lampetra japonica*) provides a mechanism for its evolutionary stability. Pflugers Arch.

[19] Xue Z, Bai J, Sun J, Wu Y, Yu SY, Guo RY, Liu X, Li QW (2011). Novel neutrophil inhibitory factor homologue in the buccal gland secretion of *Lampetra japonica*. Biol Chem.

[20] Jiang Q, Liu Y, Duan D, Gou M, Wang H, Wang J, Li Q, Xiao R (2016). Anti-angiogenic activities of CRBGP from buccal glands of lampreys (*Lampetra japonica*). Biochimie.

[21] Stancovski I, Gonen H, Orian A, Schwartz AL, Ciechanover A (1995). Degradation of the proto-oncogene product c-Fos by the ubiquitin proteolytic system in vivo and in vitro: identification and characterization of the conjugating enzymes. Mol Cell Biol.

[22] Wang Y, Köster K, Lummer M, Ragg H (2014). Origin of serpin-mediated regulation of coagulation and blood pressure. PLoS One.

[23] Magalhães GS, Lopes-Ferreira M, Junqueira-de-Azevedo IL, Spencer PJ, Araújo MS, Portaro FC, Ma L, Valente RH, Juliano L, Fox JW (2005). Natterins, a new class of proteins with kininogenase activity characterized from *Thalassophryne nattereri* fish venom. Biochimie.

[24] Tamura S, Yamakawa M, Shiomi K (2011). Purification, characterization and cDNA cloning of two natterin-like toxins from the skin secretion of oriental catfish *Plotosus lineatus*. Toxicon.

[25] Jiménez-Alcázar M, Napirei M, Panda R, Köhler EC, Kremer Hovinga JA, Mannherz HG, Peine S, Renné T, Lämmle B, Fuchs TA (2015). Impaired DNase1-mediated degradation of neutrophil extracellular traps is associated with acute thrombotic microangiopathies. J Thromb Haemost.

[26] Carlquist JF, Muhlestein JB, Anderson JL (2007). Lipoprotein-associated phospholipase A_2_: a new biomarker for cardiovascular risk assessment and potential therapeutic target. Expert Rev Mol Diagn.

[27] Dong ZD, Zhang J, Ji XS, Zhou FN, Fu Y, Chen W, Zeng YQ, Li TM, Wang H (2012). Molecular cloning, characterization and expression of cathepsin D from grass carp (*Ctenopharyngodon idella*). Fish Shellfish Immunol.

[28] Páleníková J, Lieskovská J, Langhansová H, Kotsyfakis M, Chmelař J, Kopecký J (2015). *Ixodes ricinus* salivary serpin IRS-2 affects Th17 differentiation via inhibition of the interleukin-6/STAT-3 signaling pathway. Infect Immun.

[29] Klein M, Brühl TJ, Staudt V, Reuter S, Grebe N, Gerlitzki B, Hoffmann M, Bohn T, Ulges A, Stergiou N (2015). Tick salivary sialostatin L represses the initiation of immune responses by targeting IRF4-dependent transcription in murine mast cells. J Immunol.

[30] Zavasnik-Bergant T (2008). Cystatin protease inhibitors and immune functions. Front Biosci.

[31] Dainichi T, Maekawa Y, Ishii K, Zhang T, Nashed BF, Sakai T, Takashima M, Himeno K (2001). Nippocystatin, a cysteine protease inhibitor from *Nippostrongylus brasiliensis*, inhibits antigen processing and modulates antigen-specific immune response. Infect Immun.

[32] de Faria MT, Cury-Boaventura MF, Lopes LR, da Silva JR (2014). Generation of reactive oxygen species by leukocytes of *Prochilodus lineatus*. Fish Physiol Biochem.

[33] Yang HT, Yang MC, Sun JJ, Shi XZ, Zhao XF, Wang JX (2016). Dual oxidases participate in the regulation of intestinal microbiotic homeostasis in the kuruma shrimp *Marsupenaeus japonicus*. Dev Comp Immunol.

[34] Sun J, Liu X, Li Q (2010). Molecular cloning, expression and antioxidant activity of a peroxiredoxin 2 homologue from *Lampetra japonica*. Fish Shellfish Immunol..

[35] Macey DJ, Cake MH, Potter IC (1988). Exceptional iron concentrations in larval lampreys (Geotria australis) and the activities of superoxide radical detoxifying enzymes. Biochem J.

[36] Lee JY, Cho PY, Kim TY, Kang SY, Song KY, Hong SJ (2002). Hemolytic activity and developmental expression of pore-forming peptide, clonorin. Biochem Biophys Res Commun.

[37] Don TA, Oksov Y, Lustigman S, Loukas A (2007). Saposin-like proteins from the intestine of the blood-feeding hookworm, *Ancylostoma caninum*. Parasitology.

