# Supplementary material for: Proteomic analysis of buccal gland secretion from fasting and feeding lampreys (Lampetra morii)
Source: Proteome Sci. 2018 May 22;16:9. doi: 10.1186/s12953-018-0137-5 (PMC5964706; doi:10.1186/s12953-018-0137-5)
Supplement: Supplementary file 1 — Figure S1. The distribution of mass errors is near zero and most of them are less than 20 ppm, which means the mass accuracy of the MS data fits the requirement and has a good QC validation of MS data. Figure S2. The protein composition of buccal gland secretion from lampreys fed for 10 min and 60 min was detected by 2D-PAGE. Table S1. The distribution of identified proteins on biological process (A), molecular function (B) and cellular component (C) during fasting and feeding stages. The top ten terms on biological process, molecular function, and cellular component were listed. Excel The identified protein species in the buccal gland secretion of lampreys during fasting and feeding stages in Ensembl lamprey and NCBI databases. (PDF 927 kb) [file 12953_2018_137_MOESM1_ESM.pdf]

Liaoning Normal University 8/19/2017 11:44:16 AM

m/z	S/N	Quality Fac.	Res.	Intens.	Area
721.309	6	243	9851	62.8	8.33
927.436	45	2743	16069	434	50.1
1163.570	10	1143	18969	106	14.3
1249.557	8	257	17490	84.2	12.9
1305.650	7	325	18304	72.3	11.1
1439.738	73	20669	20664	754	133
1479.723	90	27984	21981	924	160
1554.572	9	665	22192	97.8	18.1
1567.665	43	7247	22513	448	83.3
1639.855	13	1079	23257	145	28.2
1747.614	9	511	18499	97.3	25.7
1880.829	131	71013	22243	1342	365
2044.931	104	8484	23127	973	279
2524.024	18	1251	22240	127	51.0
2541.052	18	1043	22501	123	47.6

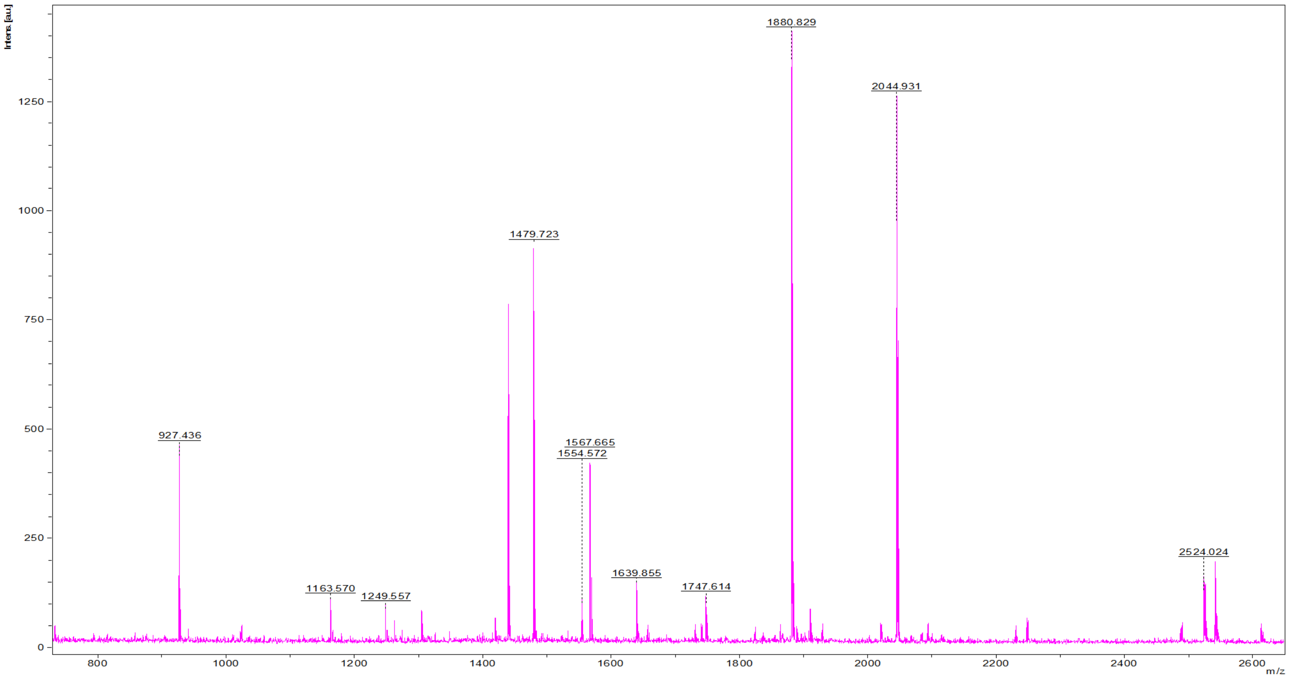
Intens. [a.u.]
1250
1000
750
500
250
0
800
1000
1200
1400
1600
1800
2000
2200
2400
2600
m/z
927.436
1163.570
1249.557
1479.723
1567.665
1554.572
1639.855
1747.614
1880.829
2044.931
2524.024

Supplementary Figure 1

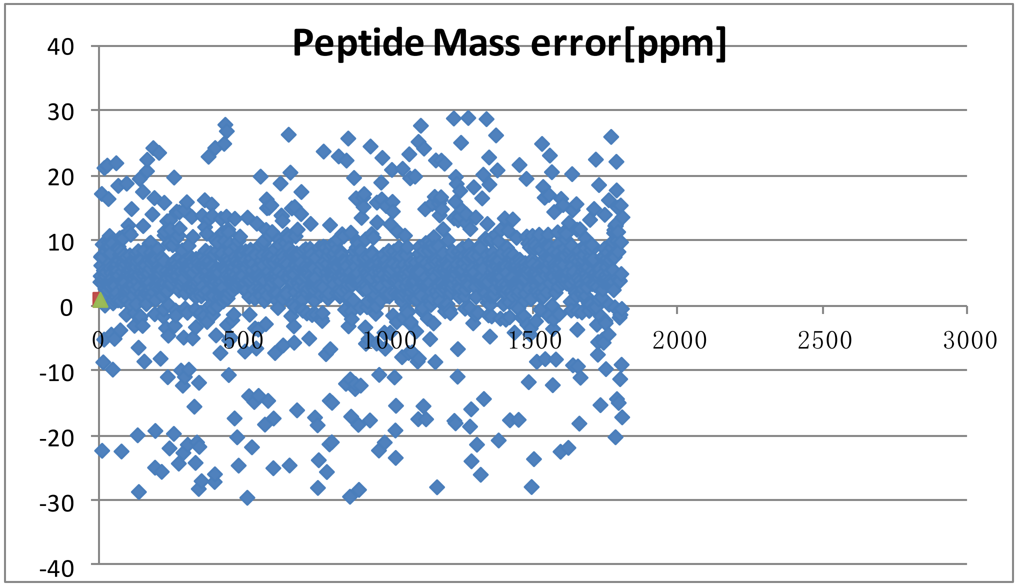

Supplementary Figure 2

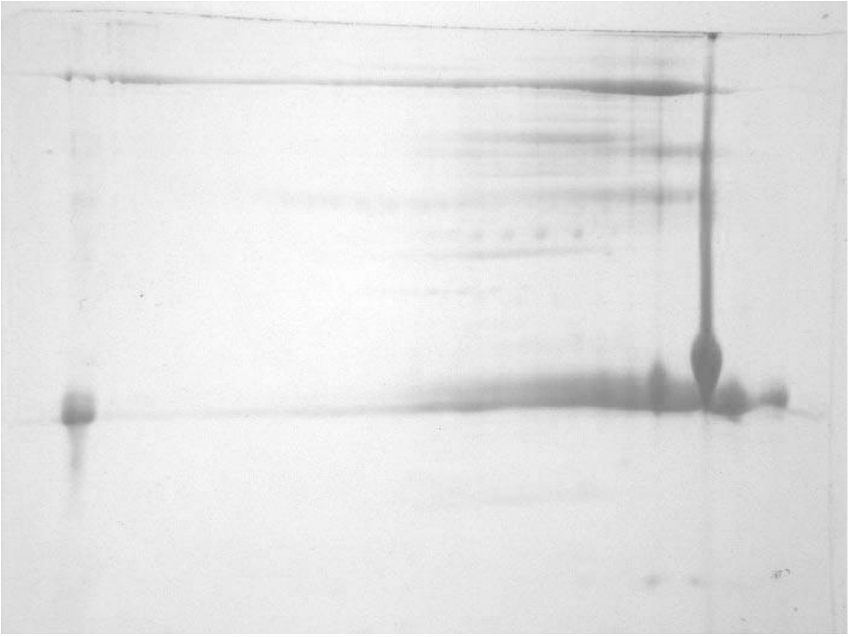

10 min

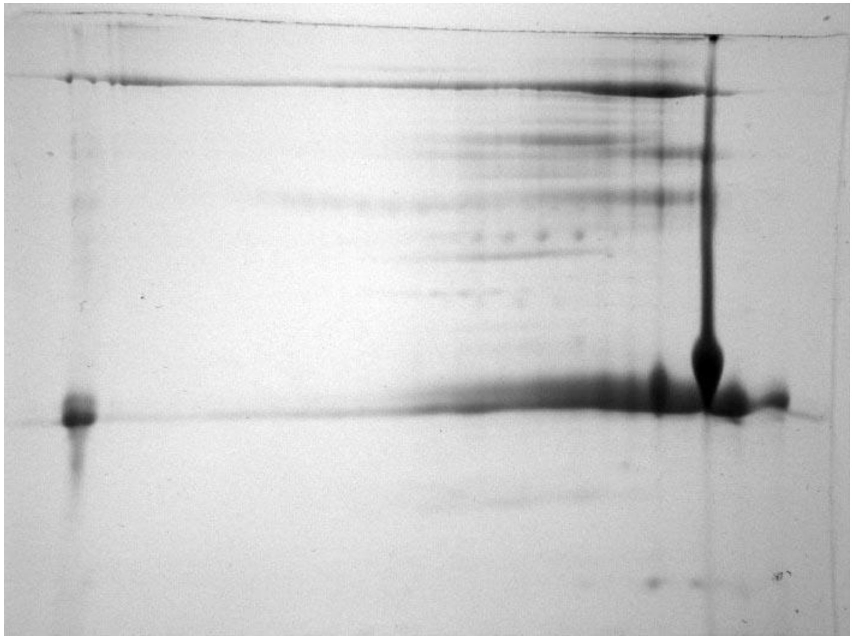

60 min

Supplementary Table 1A

Series Number	GO Term Name	Ensembl			GO Term Name	NCBI		
		0 min	10 min	1 h		0 min	10 min	1 h
1	metabolic process	27.3%	25%	24%	transport	28.5%	40%	19%
2	oxidation-reduction process	18.2%	8.3%	11%	metabolic process	14.3%	10%	18%
3	transport	45.5%	58.3%	10%	oxidation-reduction process	—	—	12%
4	signal transduction	—	—	10%	signal transduction	—	—	8%
5	proteolysis	9.1%	—	8%	glycolytic process	14.3%	20%	8%
6	small GTPase mediated signal transduction	—	—	8%	small GTPase mediated signal transduction	14.3%	—	8%
7	protein transport	—	—	8%	oxygen transport	14.3%	20%	8%
8	intracellular protein transport	—	—	7%	protein phosphorylation	14.3%	10%	7%
9	GTP catabolic process	—	—	7%	protein transport	—	—	6%
10	protein phosphorylation	—	8.3%	7%	proteolysis	—	—	6%

Supplementary Table 1B

Series Number	GO Term Name	Ensembl			GO Term Name	NCBI		
		0 min	10 min	1 h		0 min	10 min	1 h
1	protein binding	35%	38.5%	21%	nucleotide binding	10%	7.7%	20%
2	nucleotide binding	20%	7.7%	19%	ATP binding	10%	7.7%	13%
3	ATP binding	15%	15.4%	13%	transferase activity	10%	15.4%	10%
4	hydrolase activity	10%	7.7%	9%	catalytic activity	10%	30.8%	9%
5	transferase activity	—	7.7%	9%	protein binding	—	7.7%	8%
6	GTP binding	5%	—	7%	hydrolase activity	10%	—	8%
7	DNA binding	5%	—	6%	GTP binding	10%	—	8%
8	oxidoreductase activity	5%	7.7%	6%	DNA binding	—	15.4%	8%
9	structural molecule activity	—	—	5%	GTPase activity	10%	—	8%
10	metal ion binding	5%	15.4%	5%	metal ion binding	30%	15.4%	8%

Supplementary Table 1C

Series Number	GO Term Name	Ensembl			GO Term Name	NCBI		
		0 min	10 min	1 h		0 min	10 min	1 h
1	membrane	16.7%	33.3%	18%	cytoplasm	33.3%	16.7%	17%
2	cytoplasm	49.9%	—	16%	extracellular	33.3%	33.3%	16%
3	intracellular	—	—	15%	membrane	—	16.7%	15%
4	nucleus	—	—	13%	plasma membrane	—	16.7%	12%
5	intermediate filament	—	33.3%	8%	intracellular	33.3%	—	8%
6	extracellular region	16.7%	33.3%	8%	nucleus	—	8.3%	8%
7	ribisome	—	—	7%	integral component of membrane	—	8.3%	7%
8	cytoskeletion	16.7%	—	6%	mitochondrion	—	—	7%
9	mitochondrion	—	—	5%	protein complex	—	—	7%
10	chromosome	—	—	4%	chromosome	—	—	3%

Ensembl database (fasting)

Spot No.	Ensembl ID	protein	Theorical Mr/PI	Peptides matched	Sequence covered(%)	Score
1	ENSPMAP00000008289	plasma albumin prepeptide	13.3/6.64	1	6.70%	36.81
2	ENSPMAP00000005857	myoglobin	16.4/8.99	1	10.70%	98.48
3	ENSPMAP00000005910	myoglobin	16.4/9.00	5	52.70%	358.04
4	ENSPMAP00000005891	myoglobin	16.4/9.00	6	50.70%	334.62
5	ENSPMAP00000001744	myoglobin	16.4/6.14	2	19.30%	68.99
6	ENSPMAP00000008163	ARP2 actin-related protein 2a homolog (yeast)	31.2/5.90	1	2.20%	23.20
7	ENSPMAP00000005152	dihydrouridine synthase 2	31.2/9.92	1	3.60%	29.73
8	ENSPMAP00000002473	leucine-rich repeat transmembrane protein FLRT3	31.9/9.73	0	0.00%	36.11
9	ENSPMAP00000009725	coiled-coil domain containing 61	32.4/9.90	1	3.40%	24.60
11	ENSPMAP00000005159	Enoyl Coenzyme A hydratase domain containing 1	34.2/10.61	1	4.70%	29.20
10	ENSPMAP00000008415	translin-associated factor X interacting protein 1	37.2/5.40	1	1.50%	25.40
12	ENSPMAP00000001581	proteasome (prosome, macropain) 26S subunit, non-ATPase, 12	38.0/7.74	1	3.40%	31.55
13	ENSPMAP00000005508	Inter-alpha-trypsin inhibitor heavy chain H5-like protein	39.1/7.80	2	6.70%	121.65
14	ENSPMAP00000002471	leucine-rich repeat transmembrane protein	40.7/9.99	0	0.00%	36.14
15	ENSPMAP00000008937	secernin 3	45.0/6.04	1	3.60%	23.54
16	ENSPMAP00000001239	Proteasome (prosome, macropain) 26S subunit, ATPase 2	49.0/5.50	1	1.60%	25.36
17	ENSPMAP00000008411	adenylosuccinate synthase	50.8/9.20	1	1.50%	23.80
18	ENSPMAP00000009177	transient receptor potential cation channel, subfamily V, member 6	51.1/6.51	1	3.80%	28.82

19	ENSPMAP00000003132	fragile X mental retardation syndrome-related protein 1-like isoform X4	66.4/6.30	1	2.00%	23.2
20	ENSPMAP00000005361	Sidekick homolog 2b	77.3/6.12	1	1.00%	28.46
21	ENSPMAP00000000308	prominin 2(cd133/prominin)	79.0/9.21	1	1.50%	30.73
22	ENSPMAP00000009963	polymerase (DNA directed) kappa	83.8/9.10	1	0.90%	24.40
23	ENSPMAP00000003404	DEAD (Asp-Glu-Ala-Asp) box polypeptide 27	88.9/9.90	1	1.30%	25.30
24	ENSPMAP00000005992	CAP-GLY domain containing linker protein 1a	111.0/5.22	1	0.80%	37.27
25	ENSPMAP00000009438	tight junction protein	195.1/6.83	1	0.40%	24.45

NCBI database (fasting)

Spot No.	NCBI ID	protein	Theorical Mr/PI	Peptides matched	Sequence covered(%)	Score
1	gi|401705418	Hypothetical protein Y17_1183 [*Pectobacterium wasabiae* CFBP 3304]	12.1/9.70	1	8.90%	56.70
2	gi|297544925	hypothetical protein Tmath_1505 [*Thermoanaerobacter mathranii* subsp. mathranii str.	14.7/8.90	1	7.90%	72.00
3	gi|4389013	Chain A, The 2.7 Angstrom Crystal Structure Of Deoxygenated Hemoglobin [*Petromyzon marinus*]	16.3/6.20	2	20.80%	72.60
4	gi|30750169	Chain A, Crystal Structure Of Hemoglobin From River Lamprey	16.3/9.15	1	10.70%	95.36
5	gi|110825990	RecName: Full=Globin-1; AltName: Full=Globin I [*Petromyzon marinus*]	16.5/9.37	3	30.00%	98.69
6	gi|317062023	transcriptional regulator [*Fusobacterium ulcerans* ATCC 49185]	27.5/5.30	1	5.50%	57.00
7	gi|335058605	urea ABC transporter [uncultured *Acidobacteria bacterium* A3]	27.9/4.80	1	3.90%	53.60
8	gi|145046200	cysteine-rich secretory protein-buccal gland secretion [*Lethenteron camtschaticum*]	28.0/6.50	11	23.70%	425.00
9	gi|30248403	tRNA modification GTPase TrmE [*Nitrosomonas europaea* ATCC 19718]	48.6/4.80	1	2.40%	57.20
10	gi|313681747	pyruvate kinase [*Sulfuricurvum kujiense* DSM 16994]	55.0/5.49	1	2.00%	65.39
11	gi|126143340	plasma albumin [*Lethenteron camtschaticum*]	156.6/6.02	30	17.20%	1443.22

Ensembl database (feeding for 10 min)

Spot No.	Ensembl ID	protein	Theorical Mr/PI	Peptides matched	Sequence covered(%)	Score
1	ENSPMAP00000007609	lamprey keratin	16.2/5.80	1	7.50%	69.50
2	ENSPMAP00000005910	myoglobin	16.4/8.99	5	52.70%	316.39
3	ENSPMAP00000005857	myoglobin	16.4/8.99	2	23.30%	74.39
4	ENSPMAP00000008005	myoglobin	16.4/8.99	5	52.70%	265.19
5	ENSPMAP00000001744	myoglobin	16.4/6.10	1	12.70%	47.61
6	ENSPMAP00000005891	Myoglobin	16.4/8.99	6	50.70%	334.62
7	ENSPMAP00000008821	PREDICTED: uncharacterized protein C1orf50 homolog	17.0/4.80	1	9.70%	21.60
8	ENSPMAP00000003872	zgc:195245 protein FAM107B [*Rattus norvegicus*]	17.2/9.70	1	6.30%	24.14
9	ENSPMAP00000009056	PREDICTED: acid ceramidase	25.7/9.30	1	5.30%	67.80
10	ENSPMAP00000006073	Melanotransferrin, partial	28.5/5.88	1	5.60%	72.39
11	ENSPMAP00000000298	glutaminase kidney isoform, mitochondrial isoform X4	29.1/5.70	1	4.20%	24.60
12	ENSPMAP00000008259	erythrocyte band 7 integral membrane protein	31.5/7.70	1	4.90%	27.80
13	ENSPMAP00000008190	proactivator polypeptide	31.7/5.97	2	4.90%	36.03
14	ENSPMAP00000002473	leucine-rich repeat transmembrane protein FLRT3	31.9/9.70	1	4.00%	28.91
15	ENSPMAP00000008914	cytochrome P450, family 2, subfamily W, polypeptide 1	32.4/9.50	1	4.20%	24.90
16	ENSPMAP00000003316	PREDICTED: protein FAM118B isoform X3	35.5/6.24	1	2.20%	25.34
17	ENSPMAP00000005508	inter-alpha (globulin) inhibitor H2	39.1/7.84	1	3.60%	81.00
18	ENSPMAP00000002471	leucine-rich repeat transmembrane protein	40.7/10.00	1	3.20%	25.70
19	ENSPMAP00000002352	PREDICTED: arf-GAP with GTPase, ANK repeat and PH domain-containing protein 1 isoform X4	47.3/10.20	1	2.10%	22.41

20	ENSPMAP00000003404	DEAD (Asp-Glu-Ala-Asp) box polypeptide 27	88.9/9.88	1	1.30%	25.30
21	ENSPMAP00000002451	titin	2097.2/6.89	1	0.00%	33.81

NCBI database (feeding for 10 min)

Spot No.	NCBI ID	protein	Theorical Mr/PI	Peptides matched	Sequence covered(%)	Score
1	gi|30750169	Chain A, Crystal Structure Of Hemoglobin From River Lamprey	16.3/9.20	1	10.70%	72.94
2	gi|4389013	Chain A, The 2.7 Angstrom Crystal Structure Of Deoxygenated Hemoglobin From The Sea Lamprey *(Petromyzon Marinus*)	16.3/6.15	3	28.90%	212.09
3	gi|110825990	RecName: Full=Globin-1; AltName: Full=Globin I [*Petromyzon marinus*]	16.5/9.37	4	39.30%	226.19
4	gi|126143345	hemoglobin 2 [*Lethenteron camtschaticum*]	16.5/8.97	4	39.30%	175.82
5	gi|336451127	membrane protein [Idiomarina sp. A28L]	21.9/9.04	1	5.90%	54.50
6	gi|218461704	LysR family transcriptional regulator protein [*Rhizobium* etli bv. mimosae str. IE4771]	24.0/9.42	1	4.60%	57.02
7	gi|145046200	cysteine-rich secretory protein-buccal gland secretion [*Lethenteron camtschaticum*]	28.0/6.50	9	27.20%	431.37
8	gi|31581482	actin, partial [*Vanderwaltozyma polyspora*]	33.5/5.40	2	5.40%	119.00
9	gi|1703239	RecName: Full=Fructose-bisphosphate aldolase, muscle type [*Lethenteron camtschaticum*]	39.2/9.50	1	3.30%	54.20
10	gi|124027479	Cdc6-1 [*Hyperthermus butylicus* DSM 5456] [*Pyrolobus fumarii*][*Metallosphaera yellowstonensis*]	45.4/9.54	1	2.20%	50.84
11	gi|15991461	RAG2, partial [*Triakis* sp. IMCB-2001]	46.9/4.96	1	2.60%	55.61
12	gi|313681747	pyruvate kinase [*Sulfuricurvum kujiense* DSM 16994]	55.0/5.50	1	2.00%	58.33
13	gi|7331218	keratin 1 [*Homo sapiens*]	66.0/8.80	6	10.10%	399.5
14	gi|16905189	putative gag-pol precursor [*Oryza sativa Japonica* Group]	77.2/9.48	1	1.00%	70.69
15	gi|378554473	ORF1a [Astrovirus wild boar/WBAstV-1/2011/HUN]	95.0/9.19	1	1.20%	57.37
16	gi|308455280	hypothetical protein CRE_10724 [*Caenorhabditis remanei*]	111.3/5.10	1	1.00%	61.70

17	gi|330465484	hypothetical protein VAB18032_07535 [*Verrucosispora maris* AB-18-032]	123.9/5.86	1	1.40%	55.99
18	gi|126143340	plasma albumin [*Lethenteron camtschaticum*]	156.6/6.00	23	14.80%	1116.07
19	gi|386111	plasma albumin prepeptide [*Petromyzon marinus*]	158.9/6.10	1	0.80%	55.30
20	gi|147784509	hypothetical protein VITISV_030622 [*Vitis vinifera*]	159.2/10.30	1	1.00%	60.38

Ensembl database (feeding for 60 min)

Spot No.	Ensembl ID	protein	Theorical Mr/PI	Peptides matched	Sequence covered(%)	Score
1	ENSPMAP00000010145	Selenoprotein 15	6.7/5.16	2	49.20%	111.55
2	ENSPMAP00000010887	Glycine N-methyltransferase	7.7/7.56	2	42.00%	75.51
3	ENSPMAP00000001112	Diazepam binding inhibitor (GABA receptor modulator, acyl-CoA binding protein)	9.7/8.97	2	28.70%	70.81
4	ENSPMAP00000002361	Histone H4	11.0/12.03	4	40.40%	176.41
5	ENSPMAP00000009755	Cytochrome c, somatic b	11.7/10.19	1	10.50%	29.23
6	ENSPMAP00000000864	Ribosomal protein S26, like	13.3/12.19	1	12.70%	54.24
7	ENSPMAP00000008289	Uncharacterized protein plasma albumin propeptide [*Petromyzon marinus*]	13.3/6.64	1	6.70%	30.55
8	ENSPMAP00000005542	Ribosomal protein S20	13.6/10.63	3	22.50%	143.42
9	ENSPMAP00000011117	BLAST histone 2, H2a [*Danio rerio*]	13.6/11.73	2	30.20%	77.27
10	ENSPMAP00000000976	Ubiquinol-cytochrome c reductase binding protein	13.6/9.67	1	10.80%	45.99
11	ENSPMAP00000005647	Thioredoxin domain containing 17	13.6/4.62	1	15.40%	25.41
12	ENSPMAP00000006641	Uncharacterized protein sulfotransferase 6B1 [*Danio rerio*]	13.9/5.65	1	7.40%	39.98
13	ENSPMAP00000002376	BLAST PREDICTED: histone H2AX-like [*Lepisosteus oculatus*]	14.0/11.72	1	5.40%	41.48
14	ENSPMAP00000010426	Ribosomal protein L11	14.6/10.43	1	7.10%	44.28
15	ENSPMAP00000010392	Ribosomal protein S15a	14.8/10.69	1	10.80%	87.68
16	ENSPMAP00000011207	BLAST PREDICTED: histone H2A.x-like [*Oryzias latipes*]	14.9/11.18	2	11.30%	65.86
17	ENSPMAP00000004835	FK506 binding protein 2	15.4/9.74	1	10.50%	48.33

		BLAST				
18	ENSPMAP00000002367	Uncharacterized protein Histone H3c [*Culex quinquefasciatus*]	15.6/11.63	3	19.00%	123.65
19	ENSPMAP00000002363	Histone H2B	15.7/10.73	5	35.90%	230.08
20	ENSPMAP00000008419	Peptidyl arginine deiminase, type II	15.8/6.52	1	5.90%	23.34
21	ENSPMAP00000009492	Ribosomal protein L28	16.0/12.47	1	7.90%	74.38
22	ENSPMAP00000007609	Uncharacterized protein type II keratin 3 [*Lampetra fluviatilis*]	16.2/5.78	1	7.50%	60.69
23	ENSPMAP00000005873	Myoglobin	16.3/7.62	4	39.30%	282.29
24	ENSPMAP00000005857	Myoglobin	16.4/8.99	4	41.30%	322.26
25	ENSPMAP00000001744	myoglobin	16.4/6.14	2	16.00%	98.12
26	ENSPMAP00000005910	Myoglobin	16.4/8.99	5	52.70%	358.04
27	ENSPMAP00000005891	Myoglobin	16.4/8.99	6	50.70%	334.62
28	ENSPMAP00000005163	Solute carrier family 48 (heme transporter), member 1	16.4/8.99	0	0.00%	33.85
29	ENSPMAP00000001812	Cytochrome c oxidase subunit Vaa	16.5/5.10	1	6.00%	26.35
30	ENSPMAP00000003636	Calmodulin 1	16.8/3.93	2	20.10%	65.55
31	ENSPMAP00000003698	Uncharacterized protein protein kinase C epsilon type, partial [*Picoides pubescens*]	16.9/9.02	1	4.80%	26.57
32	ENSPMAP00000001490	Deoxyribonuclease I	16.9/4.94	1	8.40%	39.03
33	ENSPMAP00000007215	cystatin (leukocystatin) F	17.1/10.31	2	11.10%	74.95
34	ENSPMAP00000003872	zgc:195245 protein FAM107B [*Rattus norvegicus*]	17.2/9.70	1	6.30%	24.14
35	ENSPMAP00000006690	Annexin	17.4/4.09	2	12.80%	75.97
36	ENSPMAP00000006483	Prohibitin 2b	17.5/10.43	1	6.30%	52.61
37	ENSPMAP00000002634	Ribosomal protein S25	17.7/11.50	2	13.30%	62.75
38	ENSPMAP00000010566	Translocase of inner mitochondrial membrane 17 homolog B (yeast)	17.8/10.22	1	5.30%	25.78
39	ENSPMAP00000007019	Malate dehydrogenase 1Aa, NAD (soluble)	18.0/9.81	2	7.80%	54.95

40	ENSPMAP00000006787	Uncharacterized protein PREDICTED: legumain-like [*Oreochromis niloticu* s]	18.1/6.35	1	9.90%	26.77
41	ENSPMAP00000003464	Uncharacterized protein olfactomedin 4 precursor [*Xenopus laevis*]	18.3/5.15	1	6.00%	27.09
42	ENSPMAP00000010483	Ribosomal protein L21	18.5/11.40	1	5.60%	24.36
43	ENSPMAP00000009791	Cofilin 1	18.6/7.47	1	8.40%	39.35
44	ENSPMAP00000000207	Zgc:165571 Anterior gradient protein 3, partial [*Chlamydotis macqueenii*]	18.7/6.52	1	10.90%	63.51
45	ENSPMAP00000010481	Nucleotide binding protein-like	19.3/5.28	1	5.60%	29.19
46	ENSPMAP00000007211	Annexin	19.3/5.04	1	9.10%	49.74
47	ENSPMAP00000004452	anterior gradient homolog 2 (*Xenopus laevis*)	19.5/9.15	2	12.20%	88.67
48	ENSPMAP00000010311	Uncharacterized protein PREDICTED: keratin, type II cytoskeletal 5-like [*Alligator mississippiensis*]	20.0/4.21	2	13.10%	108.69
49	ENSPMAP00000011072	ADP-ribosylation factor 6	20.2/9.70	0	0.00%	34.62
50	ENSPMAP00000002279	ADP-ribosylation factor-like 1	20.3/7.20	1	7.70%	35.7
51	ENSPMAP00000009400	Ferritin	20.3/5.04	6	42.90%	236.04
52	ENSPMAP00000009008	Uncharacterized protein ferritin heavy chain polypeptide 1 [*Petromyzon*	20.5/5.15	1	6.20%	38.77
53	ENSPMAP00000002125	Glyoxalase 1	20.5/7.81	2	8.40%	50.59
54	ENSPMAP00000002230	ADP-ribosylation factor 3b	20.6/6.40	7	36.10%	290.98
55	ENSPMAP00000000590	Uncharacterized protein ADP-ribosylation factor 5 [*Xenopus (Silurana) tropicalis*]	20.7/6.40	2	13.80%	105.54

56	ENSPMAP00000003811	Uncharacterized protein type I keratin 18 [*Lampetra fluviatilis*]	21.0/5.22	2	8.90%	108.22
57	ENSPMAP00000008784	Proteasome (prosome, macropain) subunit, beta type, 2	21.0/6.35	1	5.90%	55.24
58	ENSPMAP00000009659	RAB2A, member RAS oncogene family	21.0/6.25	2	14.70%	129.48
59	ENSPMAP00000010221	procollagen-proline, 2-oxoglutarate 4-dioxygenase (proline 4-hydroxylase), beta polypeptide BLAST PREDICTED: protein disulfide-isomerase [*Tarsius syrichta*]	21.0/4.75	3	21.20%	124.3
60	ENSPMAP00000003782	Neuropeptide S receptor 1	21.2/9.66	1	2.70%	24.93
61	ENSPMAP00000003895	Sorcin	21.5/4.67	3	14.00%	110.96
62	ENSPMAP00000010828	Glutathione peroxidase 2 (gastrointestinal)	21.6/7.74	1	4.70%	30.25
63	ENSPMAP00000008386	peroxiredoxin 2 buccal gland secretion peroxiredoxin [*Lethenteron camtschaticum*]	21.7/7.68	3	24.90%	133.02
64	ENSPMAP00000006967	Protein phosphatase 2, regulatory subunit B, delta	22.3/5.66	1	3.60%	29.23
65	ENSPMAP00000000223	Ribosomal protein S9	22.6/10.97	4	18.00%	113.39
66	ENSPMAP00000003933	SAR1 gene homolog Ab (*S. cerevisiae*)	22.6/7.69	1	5.50%	65.74
67	ENSPMAP00000001989	RAB1A, member RAS oncogene family	22.7/5.44	1	3.90%	44.98
68	ENSPMAP00000002252	Proteasome (prosome, macropain) subunit, beta type, 3	22.8/4.75	1	6.30%	48.00
69	ENSPMAP00000010262	RAB11a, member RAS oncogene family, like	22.8/6.73	1	5.30%	25.29

70	ENSPMAP00000000757	Abhydrolase domain containing 14B	22.9/6.24	1	6.10%	46.46
71	ENSPMAP00000006576	RAB29, member RAS oncogene family	23.0/7.73	2	5.90%	42.38
72	ENSPMAP00000010385	ADP-ribosylation factor-like 6 interacting protein 1	23.2/10.00	1	3.40%	24.47
73	ENSPMAP00000001612	Peroxiredoxin 3	23.2/5.53	2	12.40%	77.47
74	ENSPMAP00000007595	Gdnf family receptor alpha 1b (glial cell line-derived neurotrophic factor,GDNF)	23.4/7.76	1	4.30%	33.88
75	ENSPMAP00000005276	Integrator complex subunit 10	23.8/6.11	1	4.30%	32.55
76	ENSPMAP00000009910	Methyltransferase like 5	23.9/6.22	1	4.70%	25.19
77	ENSPMAP00000006436	Cystathionine-beta-synthase b	24.2/9.63	1	4.10%	28.36
78	ENSPMAP00000007890	Superoxide dismutase 2, mitochondrial	25.3/9.43	2	12.10%	86.89
79	ENSPMAP00000001201	BLAST PREDICTED: LOW QUALITY PROTEIN: protein FAM161A [*Melopsittacus undulatus*]	25.4/10.76	1	2.70%	28.6
80	ENSPMAP00000002785	Adipocyte plasma membrane associated protein	25.5/6.35	1	3.10%	29.01
81	ENSPMAP00000009056	N-acylsphingosine amidohydrolase (acid ceramidase) 1b	25.7/9.33	1	5.30%	72.46
82	ENSPMAP00000009520	BLAST PREDICTED: guanine nucleotide-binding protein subunit beta-4-like [*Felis catus*]	25.8/7.85	2	7.30%	78.53
83	ENSPMAP00000006678	Tyrosine 3-monooxygenase/tryptophan 5-monooxygenase activation protein, epsilon polypeptide 1	26.6/4.60	2	8.90%	64.70

84	ENSPMAP00000000724	Triosephosphate isomerase 1b	26.8/7.86	1	6.00%	48.50
85	ENSPMAP00000006138	ADP-ribosylation factor 5	27.2/9.76	1	7.40%	29.54
86	ENSPMAP00000008362	Uncharacterized protein PREDICTED: guanine nucleotide-binding protein G(I)/G(S)/G(T) subunit beta-2 isoform X1 [*Pelodiscus sinensis*]	27.2/4.78	1	4.00%	50.90
87	ENSPMAP00000009728	BLAST PREDICTED: EGF-like repeat and discoidin I-like domain-containing protein 3, partial [*Tarsius syrichta*]	27.2/9.76	1	3.60%	28.38
88	ENSPMAP00000000605	Cathepsin Z	27.3/10.86	1	4.90%	40.35
89	ENSPMAP00000010249	Glyceraldehyde-3-phosphate dehydrogenase	27.3/9.45	1	5.90%	25.92
90	ENSPMAP00000009936	Carnosine dipeptidase 1	27.3/4.81	1	4.40%	39.73
91	ENSPMAP00000009538	CNDP dipeptidase 2 (metallopeptidase M20 family)	27.3/6.52	7	20.30%	239.79
92	ENSPMAP00000000145	Actinin, alpha 4	27.6/4.47	1	5.00%	64.66
93	ENSPMAP00000003475	syndecan binding protein (syntenin) 2	27.8/9.35	1	4.30%	29.59
94	ENSPMAP00000009298	Uncharacterized protein PREDICTED: rho-related GTP-binding protein RhoE [*Alligator sinensis*]	28.2/9.52	1	2.70%	27.25
95	ENSPMAP00000003319	Uncharacterized protein PREDICTED: protein phosphatase 1 regulatory inhibitor subunit 16B isoform X1 [*Callorhinchus*	28.3/5.86	1	2.30%	27.73

96	ENSPMAP00000005888	Uncharacterized protein PREDICTED: 14-3-3 protein gamma-1-like isoform X2 [*Xiphophorus maculatus*]	28.4/4.59	4	14.10%	157.02
97	ENSPMAP00000002193	Erythrocyte membrane protein band 4.1 like 5	28.5/9.99	1	2.90%	27.14
98	ENSPMAP00000006073	Otolith matrix protein	28.5/5.88	1	3.40%	23.70
99	ENSPMAP00000008360	ribonuclease T2	28.8/6.28	1	6.00%	54.74
100	ENSPMAP00000000478	TatD DNase domain containing 3	29.1/9.07	1	2.60%	26.74
101	ENSPMAP00000008589	Dipeptidyl-peptidase 7	29.2/5.25	1	5.30%	30.65
102	ENSPMAP00000007305	Uncharacterized protein PREDICTED: 14-3-3 protein epsilon isoform X1 [*Callorhinchus milii*]	29.4/4.59	10	32.60%	417.11
103	ENSPMAP00000008802	Uncharacterized protein PREDICTED: ADP/ATP translocase 3-like [*Haplochromis burtoni*]	29.5/10.50	1	5.70%	64.09
104	ENSPMAP00000009424	Proteasome (prosome, macropain) subunit, beta type, 5	30.0/6.27	2	8.40%	63.78
105	ENSPMAP00000003783	Voltage-dependent anion channel 3	30.2/9.03	3	12.00%	99.29
106	ENSPMAP00000003114	ankyrin repeat and SOCS box-containing 7	30.2/11.92	0	0.00%	37.92
107	ENSPMAP00000010567	Polyglutamine binding protein 1	30.2/4.98	1	2.60%	29.19
108	ENSPMAP00000006476	ATP synthase, H+ transporting, mitochondrial F1 complex, gamma polypeptide 1	30.6/10.04	1	4.00%	24.34
109	ENSPMAP00000005235	stromal cell derived factor 4	30.9/4.39	1	5.00%	86.64

110	ENSPMAP00000001842	Seven in absentia homolog 1 (*Drosophila*) BLAST PREDICTED: E3 ubiquitin-protein ligase SIAH1 isoform X2 [*Felis catus*]	31.5/6.22	0	0.00%	36.59
111	ENSPMAP00000008259	Stomatin	31.5/7.67	2	8.40%	76.90
112	ENSPMAP00000006978	VPS9 domain containing 1 (vacuolar protein sorting, May bind a Rab GTPase such as VPS21)	31.5/10.61	1	1.80%	24.20
113	ENSPMAP00000002253	sarcoglycan, gamma	31.6/5.61	1	5.40%	26.73
114	ENSPMAP00000008190	Prosaposin	31.7/5.97	2	9.10%	83.22
115	ENSPMAP00000009988	BLAST PREDICTED: sperm-associated antigen 1 [*Larimichthys crocea*]	32.0/5.22	1	4.20%	25.67
116	ENSPMAP00000005370	BLAST keratin alpha [*Lampetra fluviatilis*]	32.1/9.63	1	2.90%	67.58
117	ENSPMAP00000001254	Glutaminyl-peptide cyclotransferase	32.1/5.96	1	4.00%	32.82
118	ENSPMAP00000008914	Blast Cytochrome P450 2H2, partial [*Columba livia*]	32.4/9.49	1	4.20%	24.92
119	ENSPMAP00000008118	Zgc:136930 keratin gamma3 [*Lampetra fluviatilis*]	32.6/5.53	3	9.80%	119.76
120	ENSPMAP00000011279	Glyceraldehyde-3-phosphate dehydrogenase	32.7/7.82	2	8.50%	68.86
121	ENSPMAP00000005709	BLAST cathepsin Z precursor [*Xenopus laevis*]	32.8/5.91	1	3.40%	66.87
122	ENSPMAP00000003198	BLAST PREDICTED: netrin-G2, partial [*Pelecanus crispus*]	33.5/9.47	1	2.70%	23.21
123	ENSPMAP00000006739	Aurora kinase A	33.6/10.01	1	2.70%	24.00
124	ENSPMAP00000005779	Sulfotransferase family 5A, member	34.0/7.79	1	5.70%	37.75

125	ENSPMAP00000008676	zgc:73340 BLAST putative methyltransferase KIAA1456 homolog [*Danio rerio*]	34.0/10.96	1	2.30%	28.33
126	ENSPMAP00000006316	Angiotensin II receptor, type 1a	34.0/12.03	1	2.60%	33.85
127	ENSPMAP00000011213	Ribosomal protein, large, P0	34.2/5.72	3	11.10%	151.04
128	ENSPMAP00000011002	Uncharacterized protein PREDICTED: natterin-like protein-like [*Astyanax mexicanus*]	34.2/9.25	10	40.30%	451.92
129	ENSPMAP00000004119	Uncharacterized protein natterin-like protein [*Lethenteron camtschaticum*]	34.4/9.38	1	2.50%	81.87
130	ENSPMAP00000000696	poly(U)-specific endoribonuclease-C-like	34.4/6.17	1	2.60%	52.60
131	ENSPMAP00000007179	Pyridoxal (pyridoxine, vitamin B6) kinase b	34.5/6.01	5	15.70%	187.65
132	ENSPMAP00000004981	UDP-Gal:betaGlcNAc beta 1,4-galactosyltransferase, polypeptide 2	34.6/7.79	3	10.60%	145.13
133	ENSPMAP00000003361	Phospholipase A2, group VII (platelet-activating factor acetylhydrolase, plasma)	34.6/9.14	1	4.20%	36.47
134	ENSPMAP00000010582	2-deoxyribose-5-phosphate aldolase homolog (C. elegans)	34.6/10.03	1	5.00%	28.92
135	ENSPMAP00000001233	Uncharacterized protein natterin-like protein [*Lethenteron camtschaticum*]	34.7/6.44	3	9.80%	101.27
136	ENSPMAP00000005532	protein phosphatase 1, catalytic subunit, alpha isoform a	34.9/6.75	1	5.60%	43.28

137	ENSPMAP00000002494	Guanine nucleotide binding protein (G protein), beta polypeptide 2-like 1	35.1/7.90	1	3.50%	37.61
138	ENSPMAP00000003520	ATP synthase, H+ transporting, mitochondrial F1 complex, beta polypeptide	35.7/4.77	4	17.00%	218.19
139	ENSPMAP00000002059	Family with sequence similarity 185, member A Blast protein FAM185A isoform	35.7/9.62	0	0.00%	35.45
140	ENSPMAP00000007960	Gamma-glutamyl hydrolase (conjugase, folylpolygammaglutamyl hydrolase)	36.0/9.45	1	4.30%	44.14
141	ENSPMAP00000002676	UEV and lactate/malate dehyrogenase domains	36.4/7.02	1	4.50%	50.59
142	ENSPMAP00000006408	Serine carboxypeptidase 1	36.8/5.29	1	3.30%	57.77
143	ENSPMAP00000001757	RNA methyltransferase like 1a	37.5/10.22	1	1.80%	27.92
144	ENSPMAP00000006470	BLAST cathepsin L2 precursor [*Xenopus laevis*]	37.6/5.89	1	3.20%	43.06
145	ENSPMAP00000006718	Calcium/calmodulin-dependent protein kinase (CaM kinase) II alpha	37.7/6.19	1	3.60%	30.62
146	ENSPMAP00000010248	aminocarboxymuconate semialdehyde decarboxylase	37.8/5.78	2	8.30%	118.84
147	ENSPMAP00000002162	BLAST serpin [*Lampetra fluviatilis*]	38.2/6.19	2	7.30%	82.20
148	ENSPMAP00000001895	Leucine rich repeat containing 41	38.4/11.03	1	2.90%	37.09
149	ENSPMAP00000007456	Tubulin, alpha 7 like	38.9/5.51	2	6.60%	63.38

150	ENSPMAP00000004300	BLAST PREDICTED: D-beta-hydroxybutyrate dehydrogenase, mitochondrial-like [*Latimeria*	38.9/9.62	1	2.00%	27.01
151	ENSPMAP00000007243	Guanine nucleotide binding protein (G protein), alpha transducing activity polypeptide 2	38.9/5.12	1	3.20%	61.81
152	ENSPMAP00000001644	Calcium binding protein 39, like 1	39.1/8.97	2	6.50%	59.88
153	ENSPMAP00000005508	Inter-alpha (globulin) inhibitor H5	39.1/7.84	1	3.60%	45.09
154	ENSPMAP00000004397	Fructose-bisphosphate aldolase	39.2/9.37	10	21.00%	375.8
155	ENSPMAP00000003735	Aminoacylase 1	39.9/5.14			
156	ENSPMAP00000000210	Prolyl 4-hydroxylase, alpha polypeptide I b	40.0/4.75	1	2.20%	34.81
157	ENSPMAP00000010369	Abhydrolase domain containing 3	40.1/7.12	1	1.40%	27.34
158	ENSPMAP00000006362	ATP synthase, H+ transporting, mitochondrial F1 complex, alpha subunit 1, cardiac muscle	40.4/9.60	3	9.90%	117.99
159	ENSPMAP00000002549	ATP synthase, H+ transporting, mitochondrial F1 complex, alpha subunit 1, cardiac muscle	40.5/9.88	3	9.90%	113.68
160	ENSPMAP00000008580	Replication factor C (activator 1) 2	40.6/6.30	1	6.00%	31.89
161	ENSPMAP00000011255	G patch domain and ankyrin repeats 1	40.6/10.33	1	2.50%	25.90
162	ENSPMAP00000002471	PREDICTED: leucine-rich repeat transmembrane protein FLRT3 [*Poecilia reticulata*]	40.7/9.99	1	3.20%	28.28
163	ENSPMAP00000007858	Galactose-3-O-sulfotransferase 1	41.6/9.76	1	3.40%	32.76

164	ENSPMAP00000004567	Uncharacterized protein cytoplasmic actin [*Lethenteron camtschaticum*]	41.7/5.10	5	13.30%	210.15
165	ENSPMAP00000000348	Tubulin, beta 4B class Ivb	41.7/5.62	6	21.30%	271.04
166	ENSPMAP00000011067	Uncharacterized protein cytoplasmic actin [*Lethenteron camtschaticum*]	41.8/5.19	14	38.30%	657.59
167	ENSPMAP00000004873	Uncharacterized protein type I keratin 10 [*Lampetra fluviatilis*]	42.5/4.76	3	6.50%	120.56
168	ENSPMAP00000004933	Uncharacterized protein type II keratin 2 [*Lampetra fluviatilis*]	43.9/5.06	1	3.10%	74.18
169	ENSPMAP00000007519	Uncharacterized protein PREDICTED: zinc finger BED domain-containing protein 1-like [*Callorhinchus milii*]	44.4/6.96	1	2.30%	25.05
170	ENSPMAP00000009021	Chitinase domain containing 1	44.8/9.95	1	2.00%	29.22
171	ENSPMAP00000006869	Proteasome (prosome, macropain) 26S subunit, non-ATPase, 6	45.3/5.79	1	3.30%	30.20
172	ENSPMAP00000008862	Uncharacterized protein Protein PTHB1 [*Pelecanus crispus*]	45.4/6.68	1	2.00%	27.44
173	ENSPMAP00000010238	Thyroid hormone receptor associated protein 3b	45.7/9.81	1	1.80%	24.47
174	ENSPMAP00000002661	Uncharacterized protein type I keratin 18 [*Lampetra fluviatilis*]	45.8/5.74	1	1.70%	58.66
175	ENSPMAP00000008365	Epoxide hydrolase 2, cytoplasmic	45.8/8.79	2	8.00%	51.52
176	ENSPMAP00000007847	FERM domain containing 4Ba	45.8/9.73	1	3.00%	27.84
177	ENSPMAP00000004211	Enolase 1b, (alpha)	46.9/4.75	2	6.90%	62.76

178	ENSPMAP00000007938	Uncharacterized protein Ubc protein [*Rattus norvegicus*]	47.1/8.95	2	6.00%	64.10
179	ENSPMAP00000005835	SIL1 nucleotide exchange factor	47.5/4.92	3	9.00%	216.41
180	ENSPMAP00000006606	Echinoderm microtubule associated protein like 2	48.2/9.34	1	1.60%	25.31
181	ENSPMAP00000003032	Protein disulfide isomerase-related protein (provisional)	48.3/5.42	2	5.40%	75.62
182	ENSPMAP00000003029	N-sulfoglucosamine sulfohydrolase (sulfamidase)	48.4/5.76	1	3.10%	40.10
183	ENSPMAP00000001808	Uncharacterized protein PREDICTED: arginine-glutamic acid dipeptide repeats protein [*Capra hircus*]	48.6/9.93	1	2.10%	34.29
184	ENSPMAP00000009146	Serine/threonine kinase 25a	48.9/5.51	1	3.40%	49.34
185	ENSPMAP00000001461	Uncharacterized protein keratin type II Lfl-K 1 [*Lampetra fluviatilis*]	49.6/9.45	9	15.20%	352.61
186	ENSPMAP00000001463	Uncharacterized protein keratin type II Lfl-K 1 [*Lampetra fluviatilis*]	49.7/9.38	3	5.50%	118.52
187	ENSPMAP00000001329	Fibulin 5	49.8/5.19	1	2.40%	47.76
188	ENSPMAP00000007379	Dolichyl-diphosphooligosaccharide-protein glycosyltransferase	49.9/5.50	1	2.70%	52.09
189	ENSPMAP00000000545	Chaperonin containing TCP1, subunit 7	50.0/5.34	1	2.40%	31.22
190	ENSPMAP00000011381	Eukaryotic translation elongation factor 1 alpha 2	50.2/9.68	1	2.60%	80.51
191	ENSPMAP00000001701	Uncharacterized protein rab GDP dissociation inhibitor alpha-like	52.1/5.08	1	2.00%	48.74

192	ENSPMAP00000009361	Chromosome 9 open reading frame 72	52.9/6.52	1	2.30%	27.91
193	ENSPMAP00000007934	zgc:136930 keratin gamma3[*Lampetra fluviatilis*]	53.2/4.60	20	34.00%	1110.93
194	ENSPMAP00000000074	TNF receptor-associated factor 3	53.3/9.39	1	1.50%	25.62
195	ENSPMAP00000007275	Uncharacterized protein PREDICTED: phosphatidylinositide phosphatase SAC2-like [*Mustela putorius furo*]	53.8/6.09	1	1.40%	28.34
196	ENSPMAP00000007263	Mixed lineage kinase domain-like	53.9/6.95	1	2.60%	25.93
197	ENSPMAP00000005875	RAB guanine nucleotide exchange factor (GEF) 1 PREDICTED: rab5 GDP/GTP exchange factor [*Latimeria chalumnae*]	54.6/9.48	1	1.70%	24.75
198	ENSPMAP00000008152	Leucine rich repeat containing 6	54.8/5.74	0	0.00%	37.56
199	ENSPMAP00000002228	Eyes shut homolog (*Drosophila*)	54.8/10.32	0	0.00%	37.56
200	ENSPMAP00000007474	BLAST PREDICTED: spastin isoform X4 [*Camelus bactrianus*]	55.0/10.06	1	2.20%	24.12
201	ENSPMAP00000008501	protein disulfide isomerase family A, member 3	55.6/5.19	1	2.60%	28.66
202	ENSPMAP00000003563	Uncharacterized protein PREDICTED: collagen alpha-3(VI) chain [*Tarsius syrichta*]	57.2/10.36	7	16.60%	461.37
203	ENSPMAP00000003743	Catalase	57.2/6.55	1	2.80%	24.63
204	ENSPMAP00000008572	BLAST PREDICTED: putative aminopeptidase W07G4.4-like [*Latimeria chalumnae*]	57.3/5.97	3	6.30%	82.97

205	ENSPMAP00000011159	Uncharacterized protein cytochrome P450, family 2, subfamily AA, polypeptide 12 [*Danio rerio*]	57.6/6.63	1	1.40%	32.32
206	ENSPMAP00000010058	Uncharacterized protein type II keratin 2 [*Lampetra fluviatilis*]	57.8/8.64	8	11.00%	336.14
207	ENSPMAP00000001210	Uncharacterized protein Leucine-rich repeats and immunoglobulin-like domains protein 3, partial	58.3/9.97	2	2.60%	35.78
208	ENSPMAP00000010787	zgc:136930 keratin gamma 2 [*Lampetra fluviatilis*]	58.4/4.68	9	18.10%	491.83
209	ENSPMAP00000001207	Uncharacterized protein zinc finger and BTB domain-containing protein 39 [*Chrysochloris asiatica*]	58.4/6.03	2	3.00%	42.85
210	ENSPMAP00000000233	Pyruvate kinase, muscle, b	58.6/5.88	1	2.40%	32.76
211	ENSPMAP00000004078	Chaperonin containing TCP1, subunit 4 (delta)	58.8/9.14	1	2.40%	53.11
212	ENSPMAP00000002685	Zgc:136930 keratin gamma [*Lampetra fluviatilis*]	59.1/5.13	9	18.00%	526.10
213	ENSPMAP00000004386	Poly(A)-specific ribonuclease (deadenylation nuclease)	59.2/5.90	1	1.50%	28.90
214	ENSPMAP00000009730	Golgin A4	59.4/5.43	1	1.20%	47.83
215	ENSPMAP00000006100	Katanin p60 subunit A-like 2	60.0/8.57	1	1.50%	24.48
216	ENSPMAP00000003566	NOP56 ribonucleoprotein homolog	60.5/9.94	1	1.50%	26.15
217	ENSPMAP00000002357	MCM5 minichromosome maintenance deficient 5 (*S. cerevisiae*) DNA replication licensing factor	60.9/9.81	0	0.00%	36.69

218	ENSPMAP00000002526	CDC-like kinase 2b	61.0/9.90	0	0.00%	33.57
219	ENSPMAP00000002817	wu:fb15e04 keratin alpha [*Lampetra fluviatilis*]	61.8/5.46	15	23.90%	697.75
220	ENSPMAP00000009602	Cell division cycle 6 homolog (*S. cerevisiae*)	62.5/10.74	1	1.70%	24.97
221	ENSPMAP00000004917	Vasorin b	63.3/8.77	1	1.10%	29.72
222	ENSPMAP00000002725	Uncharacterized protein PREDICTED: receptor-type tyrosine-protein phosphatase mu, partial [*Balearica regulorum gibbericeps*]	63.7/6.97	1	1.20%	30.27
223	ENSPMAP00000008795	tumor necrosis factor receptor superfamily, member 21	65.7/10.04	1	1.80%	25.14
224	ENSPMAP00000008691	POC1 centriolar protein homolog B (*Chlamydomonas*), like	67.0/6.33	1	1.90%	37.09
225	ENSPMAP00000004130	syntaxin binding protein 1a	67.3/8.43	1	1.20%	29.83
226	ENSPMAP00000000659	Formin-like 3	67.4/5.92	1	1.20%	32.31
227	ENSPMAP00000008006	Moesin b(membrane-organizing extension spike protein)	68.6/6.37	1	1.50%	43.62
228	ENSPMAP00000009479	Immunoglobulin mu binding protein 2	70.4/7.29	1	1.40%	24.80
229	ENSPMAP00000001624	Echinoderm microtubule associated protein like 2	70.6/5.29	1	1.50%	27.90
230	ENSPMAP00000000670	chromosome 10 open reading frame	71.0/9.87	1	0.90%	31.04
231	ENSPMAP00000001070	Transcription elongation regulator 1a (CA150)	71.2/9.41	1	1.50%	32.36
232	ENSPMAP00000003688	intraflagellar transport protein 172 homolog, partial	72.0/5.81	1	1.60%	24.64
233	ENSPMAP00000005081	Heat shock protein 5	72.7/4.91	2	3.90%	86.87

234	ENSPMAP00000007015	ATP-binding cassette, sub-family G (WHITE), member 2d	73.9/9.81	1	1.50%	39.00
235	ENSPMAP00000005970	Bardet-Biedl syndrome 2	79.1/5.71	1	1.50%	26.69
236	ENSPMAP00000009078	Sperm antigen with calponin homology and coiled-coil domains 1-like a	79.9/4.64	1	0.80%	28.94
237	ENSPMAP00000009994	transferrin-a	80.3/9.47	2	3.10%	55.11
238	ENSPMAP00000003384	Heat shock protein 90, alpha (cytosolic), class A member 1, tandem duplicate 1	80.9/4.86	3	5.40%	95.52
239	ENSPMAP00000003753	Uncharacterized protein PREDICTED: collagen alpha-1(XXII) chain isoform X3 [*Xenopus (Silurana) tropicalis*]	81.2/9.55	1	1.30%	26.34
240	ENSPMAP00000003104	cysteine and glycine-rich protein 2 binding protein	83.5/7.94	1	0.80%	43.41
241	ENSPMAP00000008238	Exocyst complex component 8	84.2/8.41	1	1.00%	29.45
242	ENSPMAP00000008526	Catenin (cadherin-associated protein), beta 1, 88kDa	85.2/5.44	1	1.50%	38.10
243	ENSPMAP00000008329	Cyclin M2a Blast PREDICTED: metal transporter CNNM2 [*Latimeria chalumnae*]	85.5/6.20	1	0.80%	26.84
244	ENSPMAP00000004431	SUN domain containing ossification factor	86.0/4.96	1	0.60%	40.18
245	ENSPMAP00000006477	dystonin-like, partial [Felis catus](anchoring neural intermediate filaments to the actin cytoskeleton)	86.7/9.39	1	1.30%	38.68
246	ENSPMAP00000001105	SLIT and NTRK-like family, member	87.8/6.08	1	1.00%	27.37

247	ENSPMAP00000003404	DEAD (Asp-Glu-Ala-Asp) box polypeptide 27	88.9/9.88	1	1.30%	25.30
248	ENSPMAP00000001240	Heat shock protein 90, beta (grp94), member 1	89.3/4.60	2	2.90%	95.37
249	ENSPMAP00000005404	Protein phosphatase 1, regulatory subunit 9A	90.8/4.69	1	1.20%	27.57
250	ENSPMAP00000010708	Tubulin, gamma complex associated protein 5	92.6/10.45	0	0.00%	35.33
251	ENSPMAP00000005505	Threonyl-tRNA synthetase	93.1/9.14	1	1.00%	26.67
252	ENSPMAP00000001332	N-deacetylase/N-sulfotransferase (heparan glucosaminyl) 2a	93.7/9.84	1	0.60%	47.81
253	ENSPMAP00000004863	Helicase, POLQ-like	94.2/9.23	1	1.30%	25.32
254	ENSPMAP00000001330	Eukaryotic translation elongation factor 2, like 2	95.4/6.35	2	2.90%	121.92
255	ENSPMAP00000010154	Oxoglutarate (alpha-ketoglutarate) dehydrogenase a (lipoamide)	96.4/8.51	1	0.60%	27.14
256	ENSPMAP00000007886	Karyopherin (importin) beta 1	97.4/4.51	1	1.70%	33.54
257	ENSPMAP00000002931	prominin 1 b (CD133)	97.7/7.75	1	1.50%	58.95
258	ENSPMAP00000006065	bicaudal C homolog 1a (*Drosophila*)	100.0/10.14	1	1.10%	30.20
259	ENSPMAP00000000662	inositol polyphosphate-4-phosphatase, type Ia	102.9/9.73	1	1.00%	44.65
260	ENSPMAP00000009480	Myeloid/lymphoid or mixed-lineage leukemia (trithorax homolog, *Drosophila*)	108.7/9.72	1	0.60%	24.58
261	ENSPMAP00000000904	Dishevelled associated activator of morphogenesis 2	109.3/9.52	1	0.80%	38.67
262	ENSPMAP00000000475	Smg-5 homolog, nonsense mediated mRNA decay factor (*C. elegans*)	114.1/5.50	2	0.90%	34.29
263	ENSPMAP00000004255	Tumor protein p53 binding protein, 2	115.3/9.89	1	0.60%	33.24

264	ENSPMAP00000009524	Uncharacterized protein PREDICTED: protocadherin Fat 1-like, partial [*Pelecanus crispus*]	115.8/5.49	1	1.40%	23.55
265	ENSPMAP00000004604	RAD50 homolog (*S. cerevisiae*)	120.2/6.35	2	1.70%	31.76
266	ENSPMAP00000001007	Myosin, heavy chain 10, non-muscle	120.3/6.36	1	1.50%	29.64
267	ENSPMAP00000001305	5-oxoprolinase (ATP-hydrolysing)	127.4/6.01	1	0.60%	32.62
268	ENSPMAP00000006009	Protein tyrosine phosphatase, non-receptor type 21	127.7/9.00	1	0.50%	26.64
269	ENSPMAP00000008605	Uncharacterized protein AF4/FMR2 family member 2 isoform X2 [*Callorhinchus milii*]	130.5/10.03	0	0.00%	33.25
270	ENSPMAP00000003639	non-SMC condensin II complex, subunit G2	131.6/6.39	1	0.90%	26.76
271	ENSPMAP00000005601	Ubiquitin-conjugating enzyme E2O	133.0/5.30	1	0.70%	28.35
272	ENSPMAP00000008534	DNA-directed RNA polymerase	133.3/8.86	1	0.80%	26.10
273	ENSPMAP00000007731	Adenylate cyclase 9	135.7/8.97	1	1.30%	24.81
274	ENSPMAP00000006147	Uncharacterized protein PREDICTED: interferon-induced very large GTPase 1-like isoform X2	137.1/6.13	0	0.00%	36.17
275	ENSPMAP00000007534	Dmx-like 2	140.6/6.19	1	1.10%	27.44
276	ENSPMAP00000001026	Polybromo 1, like	142.8/7.02	1	0.60%	24.10
277	ENSPMAP00000005602	Serine/threonine kinase 36 (fused homolog, Drosophila)	144.9/5.74	2	1.40%	40.45
278	ENSPMAP00000008570	Structural maintenance of chromosomes 4	147.0/6.41	1	0.50%	46.03

279	ENSPMAP00000008843	TBC1 domain family, member 32 BLAST PREDICTED: protein broad-minded-like isoform X1 [*Latimeria chalumnae*]	147.1/6.26	0	0.00%	33.71
280	ENSPMAP00000004719	protein phosphatase Slingshot homolog 2 isoform 1	150.7/5.91	1	0.90%	27.01
281	ENSPMAP00000000569	Golgi autoantigen, golgin subfamily a,	153.3/5.31	0	0.00%	32.00
282	ENSPMAP00000004576	Rho-associated, coiled-coil containing protein kinase 1	157.1/5.78	0	0.00%	34.57
283	ENSPMAP00000000990	ATP-binding cassette, sub-family C (CFTR/MRP), member 3	166.3/7.16	1	0.30%	26.29
284	ENSPMAP00000009592	Uncharacterized protein PREDICTED: collagen alpha-3(VI) chain isoform X3 [*Chrysemys picta bellii*]	168.3/10.12	13	9.20%	734.76
285	ENSPMAP00000002751	Dual oxidase	171.9/8.69	1	0.40%	25.18
286	ENSPMAP00000008992	Tudor domain containing 6	180.5/5.66	1	0.50%	25.35
287	ENSPMAP00000010336	SET binding factor 2	181.9/9.68	1	0.30%	40.45
288	ENSPMAP00000010244	Uncharacterized protein PREDICTED: ras GTPase-activating-like protein IQGAP1 isoform X1 [*Chrysemys picta bellii*]	187.1/5.08	1	0.40%	24.1
289	ENSPMAP00000007704	Eukaryotic translation initiation factor 2 alpha kinase 4	189.2/6.12	1	0.50%	27.16
290	ENSPMAP00000002280	Sodium channel protein	189.3/9.87	1	0.50%	25.55
291	ENSPMAP00000001826	C-type lectin domain family 19, member	192.5/4.13	4	1.60%	130.76

292	ENSPMAP00000001591	Rho GTPase activating protein 23b	193.1/32.36	1	0.30%	32.36
293	ENSPMAP00000002335	Uncharacterized protein PREDICTED: nuclear pore complex protein Nup98-Nup96 isoform X2 [*Oryctolagus cuniculus*]	198.5/5.82	1	0.50%	30.97
294	ENSPMAP00000008791	Calcineurin binding protein 1	205.6/8.99	0	0.00%	34.23
295	ENSPMAP00000001899	Periaxin	211.9/5.07	3	1.80%	133.98
296	ENSPMAP00000007161	rotatin	212.2/8.66	1	0.30%	27.53
297	ENSPMAP00000003251	Tectorin alpha	218.3/5.27	2	1.00%	88.13
298	ENSPMAP00000009803	Uncharacterized protein PREDICTED: interferon-induced very large GTPase 1-like [*Chrysemys picta bellii*]	235.2/6.33	1	0.20%	36.93
299	ENSPMAP00000000553	Neuron navigator 2b	250.6/9.98	1	0.40%	50.74
300	ENSPMAP00000007926	RANBP2-like and GRIP domain containing 2	268.7/5.67	1	0.30%	28.39
301	ENSPMAP00000000599	Dynein, cytoplasmic 2, heavy chain 1	279.7/6.44	1	0.30%	25.16
302	ENSPMAP00000009365	Uncharacterized protein PREDICTED: nebulin [*Struthio camelus australis*]	335.9/9.87	1	0.30%	24.99
303	ENSPMAP00000010203	Uncharacterized protein PREDICTED: plectin-like isoform X2 [*Lepisosteus oculatus*]	336.4/5.53	1	0.30%	25.59
304	ENSPMAP00000010556	novel transcript LOW QUALITY PROTEIN: ryanodine receptor 2, partial [*Latimeria chalumnae*]	439.6/5.96	1	0.30%	24.62
305	ENSPMAP00000001794	HYDIN, axonemal central pair apparatus protein	571.0/6.28	2	0.20%	38.93

306	ENSPMAP00000002451	titin	2097.2/6.89	1	0.00%	33.81

NCBI database (feeding for 60 min)

Spot No.	NCBI ID	protein	Theorical Mr/PI	Peptides matched	Sequence covered(%)	Score
1	gi|127525	RecName: Full=Major outer membrane lipoprotein; AltName: Full=Murein-lipoprotein; Flags: Precursor	8.2/9.76	2	33.80%	94.24
2	gi|158767	ubiquitin, partial [*Drosophila melanogaster*]	8.5/7.58	2	32.90%	67.86
3	gi|16974825	Chain A, Solution Structure Of Calcium-Calmodulin N-Terminal Domain	8.5/3.84	2	39.50%	99
4	gi|3201828	major outer membrane lipoprotein I [*Pseudomonas oleovorans*]	8.8/9.30	1	12.00%	72.35
5	gi|535709	HU protein [*Pseudomonas aeruginosa*]	9.0/10.15	2	32.60%	74.04
6	gi|157368539	transcriptional regulator HU subunit alpha [*Serratia proteamaculans* 568]	9.6/10.10	3	47.80%	154.45
7	gi|51116	G-protein beta subunit [*Mus musculus*]	13.5/5.37	2	17.10%	93.39
8	gi|220950	lysozyme [synthetic construct]	14.8/10.52	1	6.90%	62.20
9	gi|3891470	Chain A, Crystal Structure Of Human Galectin-7 In Complex With Galactosamine	14.9/7.93	1	11.90%	56.04
10	gi|229751	Chain A, Structure Of Haemoglobin In The Deoxy Quaternary State With Ligand Bound At The Alpha Haems	15.1/9.50	4	31.20%	176.07
11	gi|122298	RecName: Full=Hemoglobin subunit alpha-3; AltName: Full=Alpha-3-globin; AltName: Full=Hemoglobin alpha-2 chain	15.2/10.27	2	14.20%	74.86
12	gi|9909951	ATP synthase beta subunit [*Rhizobium leguminosarum*]	15.6/6.72	1	8.80%	63.61
13	gi|4929993	Chain A, Module-Substituted Chimera Hemoglobin Beta-Alpha (F133v)	15.8/8.00	6	44.50%	254.12
14	gi|122629	RecName: Full=Hemoglobin subunit beta; AltName: Full=Beta-globin; AltName: Full=Hemoglobin beta chain	15.9/9.10	2	15.80%	112.93
15	gi|229149	hemoglobin beta	15.9/5.14	4	35.60%	103.76
16	gi|122634	RecName: Full=Hemoglobin subunit beta; AltName: Full=Beta-globin; AltName: Full=Hemoglobin beta chain	16.0/6.91	2	15.80%	95.58

17	gi|13241083	hemoglobin beta chain [*Silurus asotus*]	16.1/9.11	3	20.40%	139.66
18	gi|30750169	Chain A, Crystal Structure Of Hemoglobin From River Lamprey	16.3/9.15	5	51.00%	355.01
19	gi|157835321	Chain A, Refinement Of A Molecular Model For Lamprey Hemoglobin From Petromyzon Marinus	16.3/5.62	6	44.00%	280.07
20	gi|126143345	hemoglobin 2 [*Lethenteron camtschaticum*]	16.5/8.97	9	59.30%	425.11
21	gi|110825990	RecName: Full=Globin-1; AltName: Full=Globin I	16.5/9.37	8	59.30%	427.41
22	gi|320167770	calmodulin [*Capsaspora owczarzaki* ATCC 30864]	16.9/4.01	2	19.50%	101.51
23	gi|320583470	Nucleoside diphosphate kinase [*Ogataea parapolymorpha* DL-1]	17.1/8.98	2	17.20%	81.38
24	gi|4127588	sodium chloride cotransporter [*Bos taurus*]	17.2/10.85	1	6.80%	60.41
25	gi|188572482	putative 40S ribosomal protein RPS18 [*Phoronis muelleri*]	17.7/11.25	2	12.50%	89.81
26	gi|14719272	15 kDa cytosolic selenoprotein [*Rattus norvegicus*]	17.8/5.15	2	13.60%	75.58
27	gi|126143347	hemoglobin 4 [*Lethenteron camtschaticum*]	18.0/4.82	7	45.90%	312.12
28	gi|158451345	putative dopa decarboxylase protein [*Apha aequalis*]	18.2/9.64	2	9.20%	71.72
29	gi|152969309	peptidoglycan-associated outer membrane lipoprotein [*Klebsiella pneumoniae subsp. pneumoniae* MGH 78578]	18.9/6.37	3	20.07%	99.41
30	gi|16517086	voltage-dependent anion channel [*Gillichthys mirabilis*]	19.0/9.23	1	5.60%	62.73
31	gi|198424347	PREDICTED: similar to nucleoside diphosphate kinase [*Ciona intestinalis*]	19.3/9.69	1	6.70%	90.04
32	gi|221126247	PREDICTED: similar to ADP-Ribosylation Factor related family member (arf-3) [*Hydra magnipapillata*]	20.2/6.18	4	24.00%	147.96
33	gi|156322163	hypothetical protein NEMVEDRAFT_v1g225293 [*Nematostella vectensis*]	20.2/11.56	8	38.30%	387.84
34	gi|391344825	PREDICTED: ADP-ribosylation factor 4-like [*Metaseiulus occidentalis*]	20.4/5.44	5	26.00%	211.64
35	gi|114056	RecName: Full=Blood plasma apolipoprotein LAL2; Flags: Precursor	20.5/5.98	4	2300.00%	167.31

36	gi|1065361	Chain A, Human Adp-Ribosylation Factor 1 Complexed With Gdp, Full Length Non-Myristoylated	20.6/6.39	2	13.90%	105.54
37	gi|595280	Rap1b [*Rattus norvegicus*]	20.9/5.22	2	14.10%	131.74
38	gi|188497677	ras-related protein Rap-1A [*Taeniopygia guttata*]	21.0/5.57	3	13.60%	102.56
39	gi|194772468	GF20391 [*Drosophila ananassae*]	21.2/11.08	13	45.80%	539.87
40	gi|28630356	ribosomal protein L18 [*Petromyzon marinus*]	21.5/12.32	2	21.80%	190.52
41	gi|260944114	peroxiredoxin TSA1 [*Clavispora lusitaniae* ATCC 42720]	21.6/5.03	2	12.80%	93.84
42	gi|33863962	TPR repeat-containing protein [*Prochlorococcus marinus* str. MIT 9313]	21.8/5.38	1	3.90%	56.28
43	gi|293392580	alkyl hydroperoxide reductase [*Serratia odorifera* DSM 4582]	22.1/5.47	2	13.00%	82.22
44	gi|332264562	PREDICTED: 40S ribosomal protein S9-like [*Nomascus leucogenys*]	22.4/10.94	1	4.60%	61.57
45	gi|5146	YPT1-related protein [*Schizosaccharomyces pombe*]	23.1/5.88	2	9.20%	73.58
46	gi|2500360	RecName: Full=60S ribosomal protein L14	23.3/11.82	1	5.60%	86.03
47	gi|156555012	PREDICTED: ras-related protein Rab-2-like [*Nasonia vitripennis*]	23.5/5.97	1	6.50%	129.48
48	gi|3318722	Chain E, Leech-Derived Tryptase Inhibitor TRYPSIN COMPLEX	23.5/9.53	3	8.10%	130.02
49	gi|218296871	Nicotinate-nucleotide--dimethylbenzimidazole phosphoribosyltransferase [*Thermus aquaticus* Y51MC23]	23.8/10.42	1	4.40%	56.69
50	gi|218461704	LysR family transcriptional regulator [*Rhizobium* etli Kim 5]	24.0/9.42	1	4.60%	62.95
51	gi|225632	casein alphaS1	24.4/4.70	3	15.40%	100.52
52	gi|136429	RecName: Full=Trypsin; Flags: Precursor	24.4/7.71	2	7.80%	96.89
53	gi|2055301	proteasome subunit Y [*Lethenteron camtschaticum*]	24.9/5.80	2	10.80%	108.46
54	gi|52345832	glutathione S-transferase mu 1 [*Xenopus (Silurana) tropicalis*]	25.2/6.40	2	11.10%	67.92

55	gi|126310937	PREDICTED: glutathione S-transferase Yb-3-like isoform 1 [*Monodelphis domestica*]	25.6/5.97	1	4.60%	57.64
56	gi|530049	14-3-3 protein, partial [*Ovis aries*]	26.3/4.65	2	9.50%	171.26
57	gi|145046200	cysteine-rich secretory protein-buccal gland secretion [*Lethenteron camtschaticum*]	28.0/6.50	7	26.80%	273.53
58	gi|391340828	PREDICTED: uncharacterized protein LOC100900270 [*Metaseiulus occidentalis*]	28.1/11.38	4	13.80%	172.94
59	gi|148260723	short-chain dehydrogenase/reductase SDR [*Acidiphilium cryptum* JF-5]	28.2/10.69	2	4.50%	70.35
60	gi|118403700	tyrosine 3-monooxygenase/tryptophan 5-monooxygenase activation protein, gamma polypeptide [*Xenopus (Silurana) tropicalis*]	28.2/4.65	1	5.70%	102.83
61	gi|1197463	14-3-3 [Dictyostelium discoideum]	28.7/4.67	1	7.50%	83.31
62	gi|157128413	14-3-3 protein sigma, gamma, zeta, beta/alpha [*Aedes aegypti*]	29.4/4.59	2	12.00%	142.98
63	gi|345561222	hypothetical protein AOL_s00193g46 [*Arthrobotrys oligospora*	29.4/4.66	0	0.00%	62.08
64	gi|1405323	LMPX of lamprey [*Petromyzon marinus*]	30.0/5.99	2	8.40%	63.78
65	gi|87303693	possible ATP adenylyltransferase [*Synechococcus* sp. WH 5701]	30.8/9.90	1	2.90%	63.13
66	gi|118099442	PREDICTED: erythrocyte band 7 integral membrane protein [*Gallus gallus*]	30.8/7.63	2	13.90%	84.3
67	gi|307109985	hypothetical protein CHLNCDRAFT_142100 [*Chlorella variabilis*]	31.5/9.51	2	3.70%	65.07
68	gi|146386352	cathepsin D [*Oryctolagus cuniculus*]	31.7/6.64	2	6.50%	62.29
69	gi|239937499	adenine nucleotide translocator s254 [*Takifugu rubripes*]	33.1/10.29	3	13.00%	132.95
70	gi|313200575	hypothetical protein MPQ_0825 [*Methylovorus* sp.	33.3/10.35	1	3.10%	55.42
71	gi|374368554	ABC transporter substrate-binding protein [*Cupriavidus basilensis OR* 16]	33.7/9.52	1	2.50%	59.73

72	gi|291238366	PREDICTED: sulfotransferase family 1B, member 1-like [*Saccoglossus kowalevskii*]	35.4/6.36	1	3.60%	60.34
73	gi|270262170	glyceraldehyde-3-phosphate dehydrogenase [*Serratia odorifera* 4Rx13]	35.5/7.80	2	6.90%	143.3
74	gi|163113	guanine nucleotide-binding regulatory protein-beta-2 subunit, partial [*Bos taurus]*	35.6/6.06	2	6.10%	117.85
75	gi|147899037	malate dehydrogenase 2, NAD (mitochondrial) [*Xenopus laevis*]	35.8/9.52	2	7.10%	97.96
76	gi|25989185	glyceraldehyde 3-phosphate dehydrogenase [*Gadus morhua*]	36.1/8.73	4	18.30%	200.05
77	gi|29242787	cytosolic malate dehydrogenase [*Acipenser brevirostrum*]	36.2/7.72	3	6.90%	93.08
78	gi|13936898	glyceraldehyde phosphate dehydrogenase [*Oncorhynchus mykiss*]	36.3/7.91	4	17.70%	176.51
79	gi|462494	RecName: Full=L-lactate dehydrogenase; Short=LDH	36.4/7.02	3	11.70%	194.4
80	gi|387970584	fructose-1,6-bisphosphate aldolase [*Pseudomonas stutzeri* TS44]	38.4/5.22	1	2.30%	70.26
81	gi|1703239	RecName: Full=Fructose-bisphosphate aldolase, muscle type	39.2/9.49	10	22.60%	449.89
82	gi|120972532	alpha enolase [*Priapulus caudatus*]	39.7/5.73	2	8.70%	76.1
83	gi|2143483	hypothetical calcium-binding protein - mouse	39.8/6.83	3	9.10%	130.46
84	gi|120407311	SPARCB [*Petromyzon marinus*]	39.9/4.25	2	7.10%	56.97
85	gi|113213	RecName: Full=Actin-1	41.6/5.27	1	2.70%	61.7
86	gi|113273	RecName: Full=Actin, cytoplasmic; Flags: Precursor	41.8/5.19	10	29.50%	418.44
87	gi|374314355	tuf1 gene product [*Serratia symbiotica* str. 'Cinara cedri']	43.3/5.30	2	7.60%	103.14
88	gi|251772958	putative methyl-accepting chemotaxis protein [*Leptospirillum ferrodiazotrophum*]	43.5/5.58	2	3.70%	58.03
89	gi|395830813	PREDICTED: serpin B4 [*Otolemur garnettii*]	44.6/6.55	2	4.10%	88.77
90	gi|488838	CaBP1 [*Rattus norvegicus*]	47.2/4.80	1	3.20%	76.4
91	gi|167534702	hypothetical protein [*Monosiga brevicollis MX1*]	47.6/4.77	1	2.10%	62.07
92	gi|402501996	putative two-component sensor [*Rhodovulum* sp. PH10]	51.4/9.28	2	3.70%	61.8

93	gi|238053993	aromatic-L-amino-acid decarboxylase [*Oryzias latipes*]	54.2/6.73	2	4.80%	103.54
94	gi|402078132	hypothetical protein GGTG_10319 [*Gaeumannomyces graminis var. tritici* R3-111a-1]	54.5/5.28	1	2.30%	59.61
95	gi|313681747	pyruvate kinase [*Sulfuricurvum kujiense* DSM 16994]	55.0/5.49	1	2.00%	65.39
96	gi|47575824	mitochondrial ATP synthase beta subunit [*Xenopus (Silurana) tropicalis*]	56.2/5.27	5	12.80%	264.92
97	gi|318067305	secretory M20A dipeptidase [*Lethenteron reissneri*]	57.7/5.36	11	19.50%	449.39
98	gi|14009437	mitochondrial ATP synthase alpha-subunit [*Cyprinus carpio*]	59.5/9.82	7	15.80%	268.46
99	gi|125550515	hypothetical protein OsI_18115 [*Oryza sativa Indica* Group]	60.4/5.82	1	1.10%	60.06
100	gi|66563290	PREDICTED: t-complex protein 1 subunit gamma [*Apis mellifera*]	61.0/6.17	1	2.00%	58.77
101	gi|227538423	signal transduction histidine kinase [*Sphingobacterium spiritivorum* ATCC 33300]	61.2/4.87	1	1.70%	64.99
102	gi|83592964	hypothetical protein Rru_A1629 [*Rhodospirillum rubrum* ATCC 11170]	64.8/4.93	1	1.40%	59.13
103	gi|229552	albumin	66.1/5.71	2	4.80%	86.19
104	gi|2492797	RecName: Full=Serum albumin; Flags: Precursor	67.8/5.81	2	4.20%	83.27
105	gi|240142573	hypothetical protein MexAM1_META2p0946 [*Methylobacterium extorquens* AM1]	68.8/9.68	2	2.10%	71.6
106	gi|71987784	Protein GLY-6, isoform a [*Caenorhabditis elegans*]	71.1/9.60	1	1.80%	66.69
107	gi|113931558	ATP-binding cassette, sub-family G (WHITE), member 2 [*Xenopus (Silurana) tropicalis*]	73.5/9.64	2	3.30%	72.59
108	gi|76818381	hypothetical protein [*Burkholderia pseudomallei* 1710b]	76.1/12.39	1	1.00%	59.45
109	gi|295660730	conserved hypothetical protein [*Paracoccidioides sp* . 'lutzii' Pb01]	90.6/6.60	2	2.90%	67.22
110	gi|262195396	serine/threonine protein kinase [*Haliangium ochraceum* DSM 14365]	105.6/7.00	0	0.00%	56.82

111	gi|145479413	hypothetical protein [*Paramecium tetraurelia strain* d4-2]	117.3/9.67	2	0.80%	58.43
112	gi|357613203	Moesin [*Danaus plexippus*]	121.6/5.66	2	2.20%	72.55
113	gi|390461147	PREDICTED: uncharacterized protein LOC100406435 [*Callithrix jacchus*]	136.5/11.12	3	2.50%	102.75
114	gi|29378311	subtilisin-like protease [*Toxoplasma gondii*]	141.4/5.38	2	1.90%	57.04
115	gi|373493775	DNA-directed RNA polymerase, beta' subunit [*Eubacterium infirmum* F0142]	144.3/5.79	1	0.90%	58.19
116	gi|149068757	Rho guanine nucleotide exchange factor (GEF) 17 (predicted), isoform CRA_a [*Rattus norvegicus*]	154.6/7.98	1	0.70%	57.55
117	gi|339897150	putative pumillio protein 4 [*Leishmania infantum* JPCM5]	155.0/8.83	1	0.70%	62.2
118	gi|126143340	plasma albumin [*Lethenteron camtschaticum*]	156.6/6.02	46	26.60%	1977
119	gi|195173563	GL18391 [*Drosophila persimilis*]	167.2/6.36	1	0.50%	55.39
120	gi|322705180	Metarhizium anisopliae ARSEF 23	170.7/6.77	1	1.00%	67.7
121	gi|350427672	PREDICTED: ATP-dependent RNA helicase hrpA-like [*Bombus impatiens*]	170.8/9.55	1	0.50%	64.09
122	gi|164511455	putative non-ribosomal peptide synthetase [*Streptomyces collinus Tu* 365]	264.9/5.85	1	0.30%	57.04
123	gi|308487754	CRE-MEL-28 protein [*Caenorhabditis remanei*]	268.2/5.28	1	0.80%	58.09
124	gi|145355563	predicted protein [*Ostreococcus lucimarinus* CCE9901]	442.4/4.62	1	0.20%	59.74
125	gi|397517241	PREDICTED: neuroblast differentiation-associated protein AHNAK [*Pan paniscus*]	556.9/5.74	5	1.50%	198.13
